# Realtime monitoring of thrombus formation in vivo using a self-reporting vascular access graft

**DOI:** 10.1038/s43856-024-00436-8

**Published:** 2024-02-05

**Authors:** Daniel Hoare, David Kingsmore, Michael Holsgrove, Ewan Russell, Mahmut T. Kirimi, Jakub Czyzewski, Nosrat Mirzai, Simon Kennedy, Steven L. Neale, John R. Mercer

**Affiliations:** 1 School of Cardiovascular & Metabolic Health, University of Glasgow, Glasgow, UK; 2 Queen Elizabeth University Hospital, University of Glasgow, Glasgow, UK; 3 Bioelectronics Unit, College of Medical, Veterinary and Life Sciences, University of Glasgow, Glasgow, UK; 4 Centre for Medical and Industrial Ultrasonics, James Watt School of Engineering, University of Glasgow, Glasgow, UK

**Keywords:** Implants, End-stage renal disease

## Abstract

**Background:**

Chronic kidney disease (CKD) affects 10% of the global population costing over a hundred billion dollars per annum and leading to increased risk of cardiovascular disease. Many patients with CKD require regular haemodialyses. Synthetic arteriovenous grafts (AVG) are increasingly used to provide rapid vascular connection for dialysis. Initially, they have excellent patency rates but are critically limited by neointimal hyperplasia at the venous anastomosis, which drives subsequent thrombosis, graft failure and death.

**Methods:**

Here, we describe a system in which electrical impedance spectroscopy sensors are incorporated circumferentially into the wall of a synthetic arteriovenous graft. This is combined with an implantable radiotelemetry system for data transmission outside the patient. The system was tested using monolayers of endothelial and smooth muscle cells as well as swine blood and clots with explanted human carotid artery plaques. Sensor testing was then performed in vitro and the device was implanted in vivo in female swine.

**Results:**

The device can wirelessly report the accumulation of biological material, both cells and blood. Differences are also detected when comparing controls with pathological atheroma. In swine differences between blockage formation in a graft were remotely obtained and wireless reported.

**Conclusions:**

Combining electrical impedance spectroscopy and an implantable radiotelemetry system enables graft surveillance. This has the potential to be used for early detection of venous stenosis and blood clot formation in real-time in vivo. In principle, the concept could apply to other cardiovascular diseases and vascular implantable devices.

## Plain language summary

Chronic kidney disease is common throughout the world and required treatments are expensive. People with chronic kidney disease require frequent blood dialysis treatment to filter their blood and remove waste products and toxic substances circulating in the blood. For some patients, implantable tubular structures called AV grafts are used for providing access to dialysis. These grafts frequently block sometimes without warning leading to patients not being able to undergo dialysis. Through a series of laboratory experiments looking at cells that block the graft, fatty deposits and blood clots, we evaluated whether sensors could detect blockages in an AV graft. We also tested the device in an animal model. From these results we were able to show that our device could detect blockages within a graft. In the future we hope that introduction to the clinic of an optimized version of our device will reduce costs to healthcare systems and improve patient outcomes.

## Abstract (web summary)

Hoare et al. evaluate whether a self-reporting arteriovenous graft can be used to detect venous stenosis and thrombus formation using in silico, cell culture and pre-clinical swine models. The graft detects differences in blood flow in a swine model following heparin administration.

## Introduction

Chronic kidney disease (CKD) affects over 843.6 million people worldwide accounting for roughly 10% of the world’s population^1,2^. It is predicted to be the 5th largest cause of mortality by 2040^3^. Within the UK there are currently 63,162 patients with CKD, while in the USA 726,331 patients suffer from the disease^3–5^. However, the prevalence of the disease is the largest in those countries with low to middle income^1^. Treating this disease consumes a significant proportion of global healthcare budgets. For example, the National Health Service (NHS) in England spent £1.45 billion in 2009–10^5^.

**Fig. 1:**
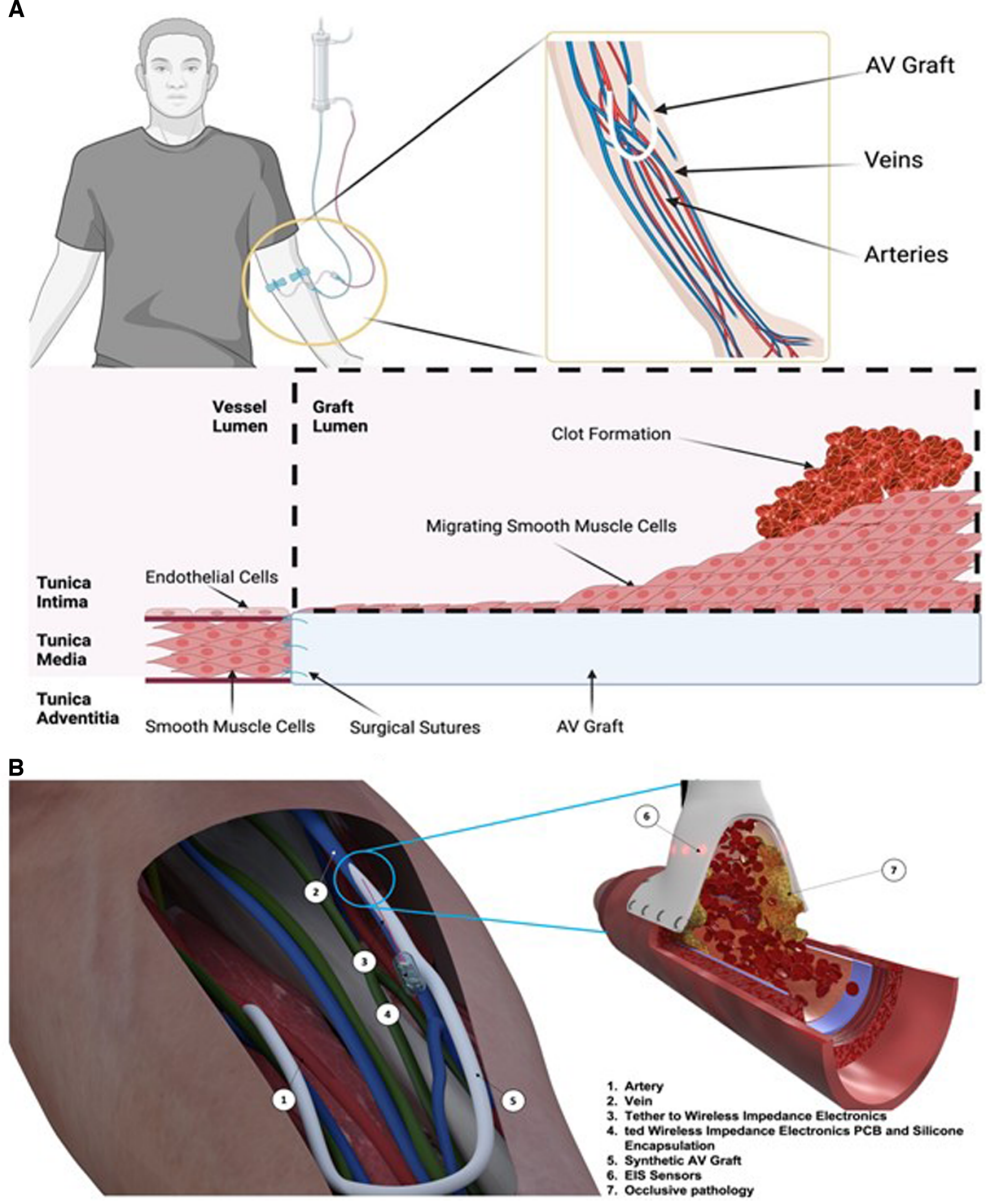
Human dialysis and self-reporting AV graft solution. Arteriovenous grafts (AV grafts) allow for early cannulation to treat patients with end-stage kidney disease. To implant an arteriovenous graft a vascular surgeon joins an artery to a vein in the upper forearm, in this case, a horseshoe join is shown (**A**). The suturing of the synthetic graft to the blood vessels causes endothelial damage leading to the formation of neointimal hyperplasia through the migration and proliferation of smooth muscle cells. This leads to venous stenosis which initiates the clotting off of the graft over time, reducing blood flow through the arteriovenous graft. Panel (**B**) depicts the arteriovenous graft (1, 2, 5) in place in a forearm with a self-reporting impedance analyser (4) and sensor interconnect (3). The right panel shows the anastomosis site with flow of blood and circumferential sensor location (6) and a representative occlusion (7).

In the United States of America (USA), Medicare’s expenditure for CKD and end-stage kidney disease (ESKD) was over $114 billion^4^. As a patient’s disease progresses the economic expenditure per patient dramatically increases due to the additional care provisions required^6^. End-stage kidney disease occurs when the functional capacity of the kidney is insufficient to filter out the toxins in the blood^7^. Multiple factors are responsible such as diabetes, hypertension, infection or acute vascular injury which reduces blood supply to the kidneys^8,9^. Over time this can lead to micro- and macrovascular organ damage as well as systemic vascular damage^10^. This perturbs hormonal and cytokine homoeostasis in the kidney, promoting inflammation that drives hypertension, endothelial dysfunction and subsequently systemic cardiovascular disease^7^. Treatments for CKD can slow progression but none can reverse the process. In addition, there are only a limited number of organs for transplant. This means the patient outlook is poor and the mainstay of treatment for ESKD relies on haemodialysis^11^. Here the patient’s blood is filtered through an external machine which is a dialyzer where fluids and electrolytes are balanced, the toxins are removed through a semipermeable membrane, before the cleaned blood is returned to the patient (see Fig. 1a^12^). CKD patients will often require this treatment multiple times a week, and as such native vessels become easily damaged from repeated cannulation to the point that vascular access (VA) deteriorates, leading to patient mortality. The unadjusted 5-year survival of ESKD patients on kidney replacement therapy was 41% in the United States and 48% in Europe between 2003 and 2016^13^. Even with a high proportion of expenditure on treatment for these patients, mortality remains high. For haemodialysis, there are two main types of VA: either arteriovenous fistulas (AVFs) or arteriovenous grafts (AVGs). Both allow for the formation of an easy-to-access repeatable cannulation site however the approaches are different. For an AVF a high-pressure artery is connected to a low-pressure vein to produce a hybrid vessel with a large surface area for repeated cannulation^14^. AVF has decreased rates of infection and decreased mortality with longer secondary patency when compared to AVG. However, a recent paradigm change from “fistula first” in vascular access has changed this concept with the type of access used dependent on the patient’s overall goals and life plan, with AVG becoming an increasingly popular alternative. The AVG is a synthetic graft to joins the artery and the vein^7^. Traditionally the fistula’s first approach to haemodialysis has been an AVF in all patients instead of an AVG due to the low maintenance of an AVF over its lifetime. However, the AVG has several advantages; one such is that the patient can receive haemodialysis treatment immediately following surgery whereas an AVF requires weeks for the native vessels to mature into an AVF^15^. Additionally, there is a much higher procedural success rate in haemodialysis where access is via AVGs. However, this is met with longer-term costs.

Both AVFs and AVGs suffer from high complication rates caused by loss of primary patency. This occurs due to the dual threat of vascular stenosis (VS) followed by thrombosis^16^. The leading limitation of the wider use of AVG is the rate of re-intervention for VS, with up to 50% requiring intervention at 1 year^3–5^. Within 1-year post-implantation, thrombosis had occurred in 69% of AVFs and 50% of AVGs^17,18^. Moreover, primary patency, the term used for AVGs needing reintervention after implant for AVGs was 38.2% and 24.6% in year 1 and year 2, respectively^19^. VS is caused by neointimal hyperplasia and is primarily found at the venous anastomosis site of an AVG or AVF^17^. Neointimal hyperplasia is initiated through the damage to the vessel wall when the access vessel or graft is sutured in place causing endothelial damage, (Fig. 1b). This in turn causes an inflammatory cascade that induces the excessive proliferation of vascular smooth muscle cells (VSMCs)^20^. This proliferation decreases the luminal area thus restricting the flow of blood into the graft which leads to reduced efficiency in the haemodialysis treatment. A 25% reduction in flow from baseline will have a significant impact on the volume of fluid that can be exchanged during haemodialysis for the patient^21^. Compounding this is that neointimal hyperplasia creates a prothrombotic environment once more reducing the cross-sectional area and reducing flow^17^. In summary, VS is silent and not preventable; reintervention of the AVG is the only candidate to save a patient’s VA.

VS is the leading cause of graft failure followed by thrombosis, both reduce the longevity of AVGs as well as lead to reintervention^5,17^. Ultrasound surveillance and reintervention caused by these conditions are associated with significant costs^5^. Ultrasound is a costly process for detecting complications and can only detect large reductions in AVG flow within a hospital setting and at the specific time point of investigation. It does not provide temporal trends and is a suboptimal solution for the monitoring of AVGs. Moreover, VS which later manifests as thrombosis is not preventable with current technology on the market^21^. Therefore, with strong evidence that early recognition allows pre-emptive treatment—rhetoric throughout modern healthcare—a graft that can track the earliest signs of VS occlusion over time could save costs, reduce mortality and increase the longevity of the AVG, thus improving patient wellbeing.

**Fig. 2:**
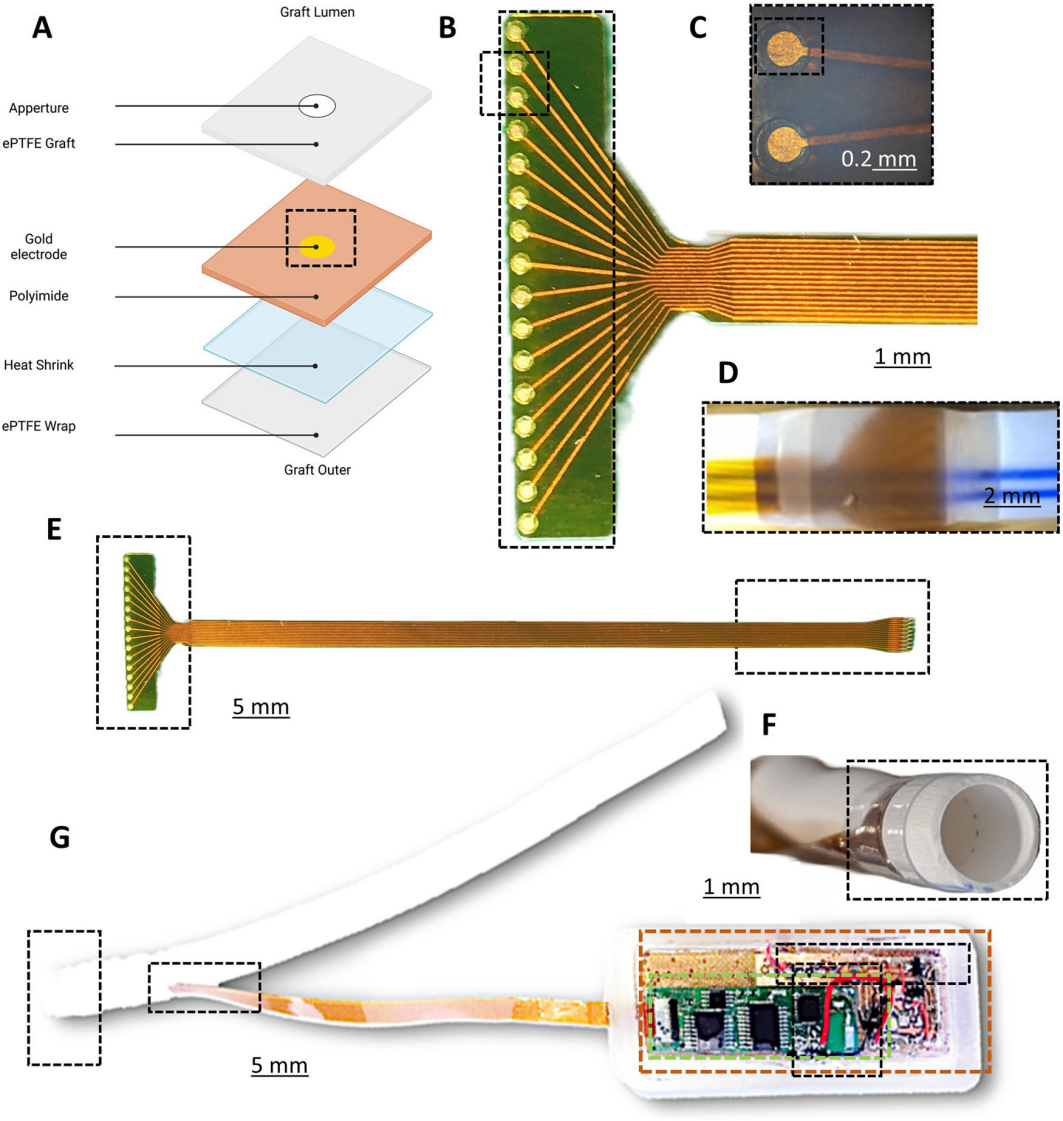
Device fabrication and integration of a self-monitoring and reporting graft. **A** Schematic of the graft fabrication layers. **B** Polyimide 16 gold electrode array. Panel **C** Expanded view of the sensing array (×10). **D** Electrode array in situ, illuminated intraluminally to highlight the sensor and sensing apertures. **E** Full-length array highlighting proximal sensor and distal connection to telemetry. **F** A prototype device with intraluminal circumferential sensor apertures on a single plane on the inside of the graft and the sensor held in place with clear heat PTFE shrink. **G** Prototype device attached to wireless impedance electronics (highlighted green) power source (highlighted gold), magnetic reed switch as used for in vivo studies.

One avenue for the remote and early detection of VS is the use of a self-reporting vascular graft. Electrical impedance spectroscopy (EIS), though an established technique, has not previously been applied to vascular pathologies. This is due to the distinct physical differences between cells and larger biological tissues namely the cell shape, conductivity of the membranes and the surrounding medium conductivity. With an alternating current, the frequency of the current affects the path it will take in the presence of matter. Through two electrodes with cells or biological matter in situ, the current at low frequency will pass paracellularly, around the cell, while at higher frequencies it will pass transcellular, through the cell. Due to the non-destructive nature of EIS temporal trends can be assessed by sensors adding an additional element to pathology monitoring. For example, the use of alternating currents (AC) for the monitoring of cell adhesion, growth and migration is well documented in vitro^22–34^. Additionally, the characterization of larger tissue structures such as blood clots through EIS is possible^35^. However, applications for in vivo remote monitoring by EIS are not established. To this avail, we show the preclinical development of a graft with circumferential EIS sensors integrated in-between the layers of the graft wall (see Figs. 1 and 2). An implantable device with wireless impedance electronics will compute, format and relay the impedance information wirelessly to outside the body (Fig. 1). Being able to detect VS in real-time without additional hospital visits would provide a key diagnostic and surveillance tool in a physician’s arsenal for a patient-centred pathway. Combining circumferential EIS sensors into the graft of a haemodialysis patient would allow physicians to remotely monitor their patient’s graft patency allowing for better management of an already complex condition.

The AVG is particularly suitable for the integration of EIS sensors into the implant. Previously, our group has developed an EIS sensor for the remote monitoring of neointimal hyperplasia in stents^36–38^. This was a rigid substrate, with interdigitated electrodes that could read a single pair of electrodes. Here we present an application for EIS sensors in an AVG. We present a series of in vitro experiments to simulate processes found during graft failure. We then successfully validate the wireless transfer of data about the in situ vessel conditions from a clinically relevant in vivo preclinical model.

## Methods

### Human ethics

We were granted ethics approval to undergo our study by the West of Scotland Research Ethics Service. The human carotid endarterectomy tissue samples were used under license from the NHSGGC Biorepository and Pathology Services (REC 10/S0704/60). Informed patient consent was obtained for these samples to be used for research.

### Animal model

Preclinical trials were conducted in compliance with the 1986 Scientific Procedures Act and approved by the United Kingdom Home Office under license number PP1529920. Wild-type large white sows (~65 kg) were used for all procedures due to the appropriate pressures and vessels that are applicable to an average human forearm. Animals were humanly sacrificed by an approved Schedule 1 method.

### Graft fabrication

The 16-electrode arrays were fabricated using a commercially available expanded polytetrafluoroethylene (ePTFE) graft (Bard, USA). Briefly, an ePTFE graft with a 6 mm internal diameter and outer wall diameter of 7.2 mm had 16 equally spaced 300-micron apertures punched through the wall (Syne, USA). The array was exactly aligned to these holes, heat shrunk in place (FEP, Heat Shrink, Zeus) and fixed in place through a silicone epoxy (ADH1 M200 Silicone Adhesive, Elkem). After sensor mounting, an ePTFE membrane was wrapped to fully seal the device in situ (PTFE Tape, TaegaSeal). Gaps were backfilled with epoxy to ensure no leaking around the sensor exit (see Fig. 2A–G).

### Sensor fabrication

Two multi-electrode arrays were fabricated by Merlin Flex PCB Ltd. (UK). The arrays consisted of a 32-electrode array with 100 μm diameter electrodes and a 16-electrode array with 500 μm diameter electrodes. These were constructed on polyimide in an adhesive layering. A rigid silicon stiffener was located at the tail of the array where the electrodes were individually addressed. Figure 2E shows a fabricated 16-electrode array with 2 electrodes of a sensing pair shown in Fig. 2C. Figure 2D shows trans-luminal illumination of the sensor in place on the graft. The transluminal holes for the sensor are shown in Fig. 2F.

### Wireless impedance electronics design

The wireless impedance electronics were adapted from our previously described circuitry^37^. The design was modified to include a 32-channel multiplexer to the electrodes, these were split into two banks A and B of 8 electrodes. Addressing any 2 electrodes completes the circuit to form a sensor pair, one from bank A and one from bank B. This gives a total combination of 64 unique spatial distinct sensors. To limit current consumption and data handling within the device, only 32 are used. A magnetically actuated power switch is used to activate and deactivate the device while for on-bench testing a wired switch is used for ease of handling (Fig. 2G black hatching). An external base station is used to communicate with the implantable electronics. Data transfer is achieved through a tuned antenna on both electronic devices which may also be used for power transfer potentially, in the case of this paper a battery is used. A microcontroller in conjunction with a multiplexer can selectively introduce electrodes to the circuit allowing the impedance to be measured.

### Encapsulation of the device

The Medical Device Manufacturing Centre of Heriot-Watt University (Edinburgh, UK) fabricated a silicone encapsulant capable of 28 days of emersion in an aqueous environment. The outer encapsulant measured ~25 mm × 75 mm × 25 mm which created a minimum thickness around the PCB and battery of 4 mm. The sensor exits through the side of the encapsulate and is backfilled with silicone epoxy to ensure water tightness in this area (see Fig. 2G).

### Cell culture and tissue handling

For the testing of the early warning system in vitro *vessel-derived* cells were used. Mouse aorta smooth muscle cells (MASMC), from an adherent transgenic mouse model^39^, and previously immortalized mouse endothelial cells (MECs)^40^, were incubated at 37 °C with 5% carbon dioxide at 95% relative humidity. Dulbecco’s Modified Eagles Medium (DMEM) containing HEPES buffer (Gibco, UK)) supplemented with 10% foetal bovine serum, 1 mL per 100 mL penicillin/streptomycin and l-glutamine was used as a control and for the culture of the cells. Each cellular experiment was seeded at 250,000 cells/mL, and a total volume of 2 mL was used for each chamber. The control was 2 mL of media only. Cells are regularly tested for mycoplasma (Promocell PK-CA91-1048).

### Monolayer staining

CellTraceTM Calcein Red-Orange, AM (C34851, Thermo Fisher), which fluoresces red was used for MASMC and Calcein, AM (C1430, Thermo Fisher), which fluoresces green was used for MECs in monolayers. Each dye was handled as per the companies’ instructions. Briefly, the monolayer was washed with PBS twice and dyes suspended in DMEM were added to the culture chamber and incubated for 40 min. Finally, cells were washed with PBS and imaged under an upright microscope (Olympus BX40).

### Blood and plaque investigations

Heparinised swine blood and clotted blood were sourced from the University of Glasgow Veterinarian School. Blood was warmed to 37 °C in a temperature-controlled water bath. Clot was warmed to 37 °C and dissected into weight-controlled samples of 10 g human carotid artery specimens, as described in our prior work^37^.

### Experimental setup

A commercially available LCR (Hikoi IM3536, Japan) was connected to the SMART Graft through a surface-mounted connector and breakout board. Here pairs of electrodes could be manually selected with the frequency range, number of readings and interval between readings programmed. The wires were insulated from the incubator where the device was placed for the testing duration.

### Numerical simulations and modelling

A 3D model of the graft was constructed using Fusion 360 (AutoDesk, USA) before being imported and converted to a Multiphysics simulation using COMSOL (COMSOL version 5.5, Sweden). A cut plane was placed through the centre of the electrodes which can be seen around the circumference of the vessel as crenelations. We produced a 2D plane in which Maxwell’s equations could be approximated across nodes generated by the solver-generated mesh. The simulation parameters are shown in Table 1.

**Table 1:** Parameters for numerical simulations

Object	Diameter (mm)	Width (mm)	Height (mm)	Permittivity	Conductivity (S/m)	Reference
ePTFE Graft	6–7.2	–	–	1	6.67 × 10^−16^	
Gold Electrode	–	0.3	0.125	1	4.56 × 10^7^	
Emboli	1	–	–	2000	4.2 × 10^−4^	^41^
Clot	–	~3.5	~1.8	2000	4.2 × 10^−4^	^41^
Blood	–	–	–	5197	70.08 × 10^−2^	^42^

### Statistics and reproducibility

Data were collected and stored using Microsoft Office 365. Statistical analysis was performed via GraphPad Prism 9. Data from cell culture experiments were analysed using a two-way repeated measures ANOVA to test for covariants with multiple comparisons. Such as for the effects of frequency and cell growth or blood clotting on impedance. A Welch’s *T*-test was performed to assess the paired measurements.

### Reporting summary

Further information on research design is available in the Nature Portfolio Reporting Summary linked to this article.

## Results

**Fig. 3:**
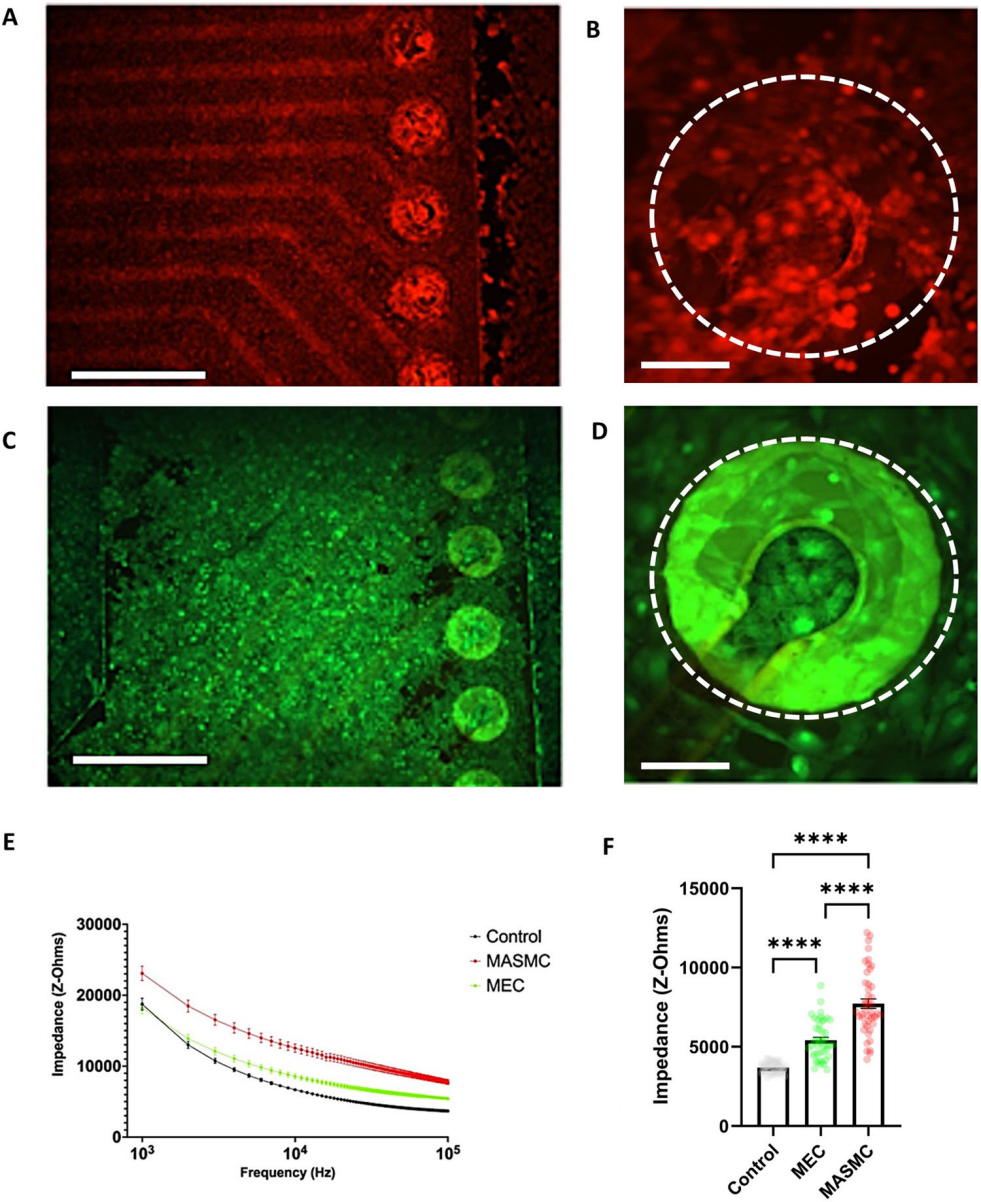
Growth of cell monolayers across the multi-electrode array. **A** The sensing array with a monolayer of MASMCs at ×4 (calcein red stain) **B** with a single electrode imaged at ×10 magnification. **C** shows the sensing array with a monolayer of MECs at ×4 with **D** a single electrode imaged at ×10 magnification (calcian green stain). **E** Show frequency spectrums of MASMCs versus MECs versus culture media controls. **F** Compares the control group, MASMCs and MECs at 100 kHz. Panels **A** and **C** scale bar represent 10 mm and **B** and **D** represent 100 microns (*N* = 3 independent biological repeats) mean ± SEM one way ANOVA, *F*-value = 97.02, degrees of freedom = 2. ****P < 0.0001. Exact *P*-values are in the Supplementary Data.

Initial testing of devices was performed in vitro using monolayers of cultured primary cells with a minimum of three biological replicates, with three technical replicates. Statistical tests are specific for each experiment but where an increase or decrease in the magnitude of change was possible then two-sided tests were used. A biocompatible cell chamber was 3D printed and secured over the polyimide sensor. Chambers were seeded with either 150,000 mouse aorta smooth muscle cells (MASMCs) or mouse endothelial cells (MECs) and incubated for 24 h Dulbecco’s modified Eagles medium (DMEM) and (4-(2-hydroxyethyl)−1-piperazineethanesulfonic acid (HEPES)-buffered culture media. An experimental control of media only was used and maintained at 37 °C as a comparison. Neointimal hyperplasia was first characterized by the migration of vascular smooth muscle cells from the vessel wall where the graft had been sutured or anastomosed to the vein or artery. Conversely, the sutured graft can undergo re-endothelisation where the endothelial cells become polarized to the direction of flow which helps to maintain good graft patency. Therefore, it is important to quantify cell monolayers’ contribution to the sensor impedance. Cell monolayers were routinely measured in triplicate and were then stained with red and green calcein fluorescent dyes (see the ”Methods” section). An intact MASMC monolayer can be observed across the whole sensor in Fig. 3A with a representative electrode in Fig. 3B also covered. The same is true for the MECs in Fig. 3C, D. During the dying stage, part of the monolayer can become detached, hence the importance of taking repeated impedance measurements. In Fig. 3E the mean electrode impedance across 1–100 kHz is shown. For a single frequency, 100 kHz, the comparison was made through a one-way analysis of variance (ANOVA) in Fig. 3F. A significant difference can be observed between the impedance measured in the control compared to both cell types and there is also a significant difference between the two cell types. Extrapolating this data suggests that when integrated into the graft wall this device could measure both the magnitude and phase change in impedance for multiple frequencies. This could discern the difference between the early stage of neo-intimal hyperplasia and that of endothelisation. COMSOL modelling was then used to corroborate this phenomenon in silico.

**Fig. 4:**
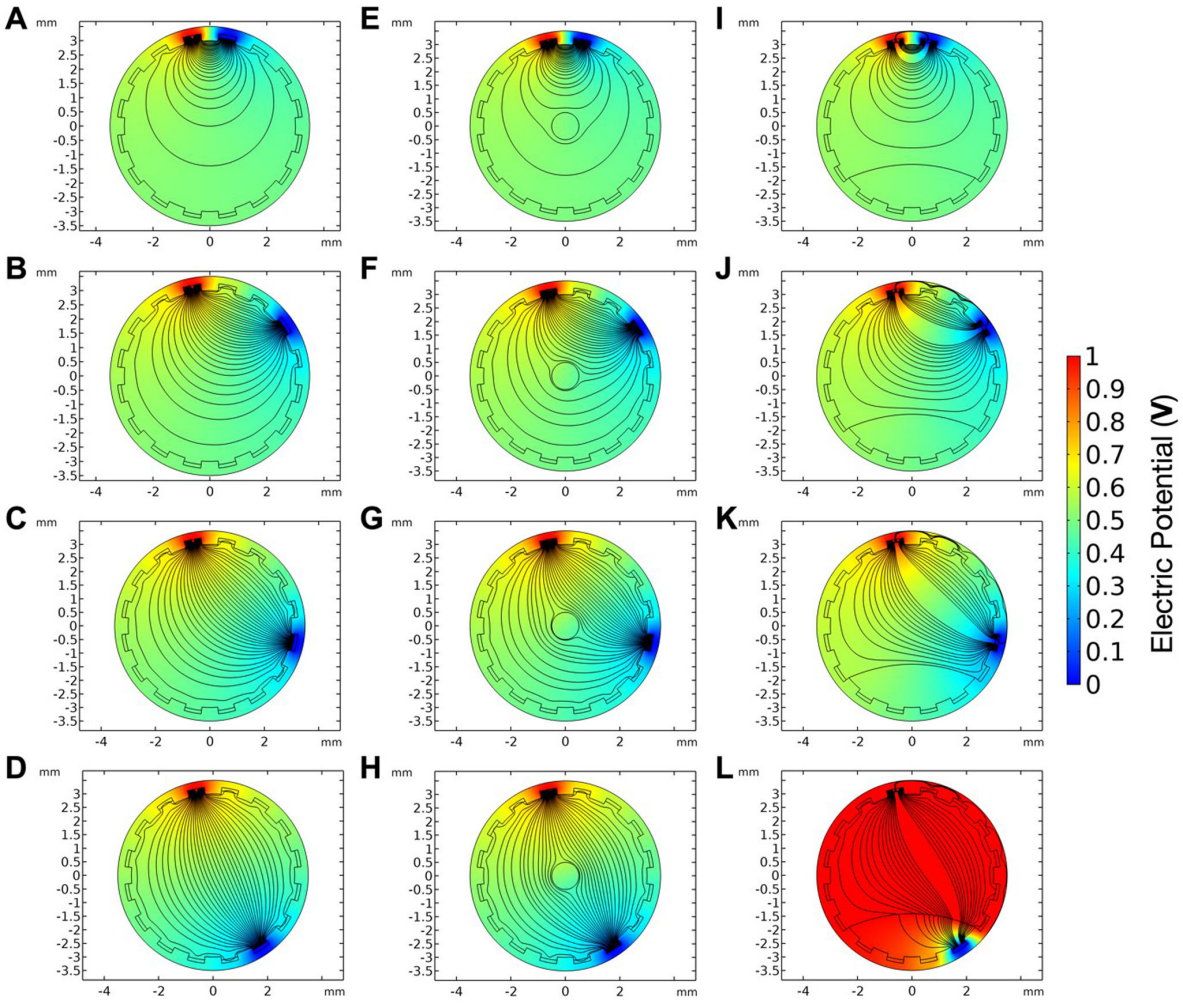
Numerical simulations of the graft under optimal and pathological conditions. In a patent graft lumen the electrical field, black streamlines, is unobstructed between the electrodes, **A**–**D** shows this with a selection of electrodes being activated. **E**–**H** show emboli in the centre of the vessel, and thrombus **I**–**L** with the thrombus at the bottom of the vessel, respectively obstruct and divert the electric field increasing the impedance measured.

Using a numerical finite element simulation, (COMSOL v5.5, Sweden https://www.comsol.com/multiphysics), the electric potential between the electrodes, colour gradient, and field lines, black streamlines, were visualized (Fig. 4). This is done by taking the transverse plane of the graft where the sensors are located (see Fig. 1 concept). Conditions investigated were; no occlusion, emboli in the lumen and thrombus formation, respectively (Fig. 4A–D, E–H and I–L). In turn, each pairing was tested with a single current injecting terminal and selected pairings depicted in Fig. 4. Where only blood is present unobstructed field lines indicate current flowing normally between electrode pairs while the electric potential drops from high at one electrode to low at the other. In Fig. 4I–L a thrombus is blocking the lower ~20% of the graft. With thrombi and emboli being rich in red blood cells and fibrin, the binding agent of a blood clot, the conductivity and permittivity of the area are lower than that of the surrounding blood^41–44^. As such when the field interacts with the different mediums the electric field lines can be seen to be diverted around the less-conductive environment of the emboli and clot. In Fig. 4E–I, the proximity of the neighbouring electrodes and their distance from the obstruction means there is little effect on the potential distribution and hence the current that flows. While through Fig. 4J–L one observes a progressive restriction of the field lines as the electrodes move from a perpendicular to a parallel arrangement denoted by the electric field lines constrained to the open lumen. As such the overall impedance the sensor measures will increase as the more conductive blood is replaced by the less conductive occlusive material reducing the ease at which the current may flow. With the emboli depicted the pathway is only marginally impacted compared to the thrombus. In particular, Fig. 4L shows the dramatic effect caused by a thrombus directly on one of the electrodes being activated therefore aggregated thrombus will cause larger impedance changes than free-flowing emboli.

**Fig. 5:**
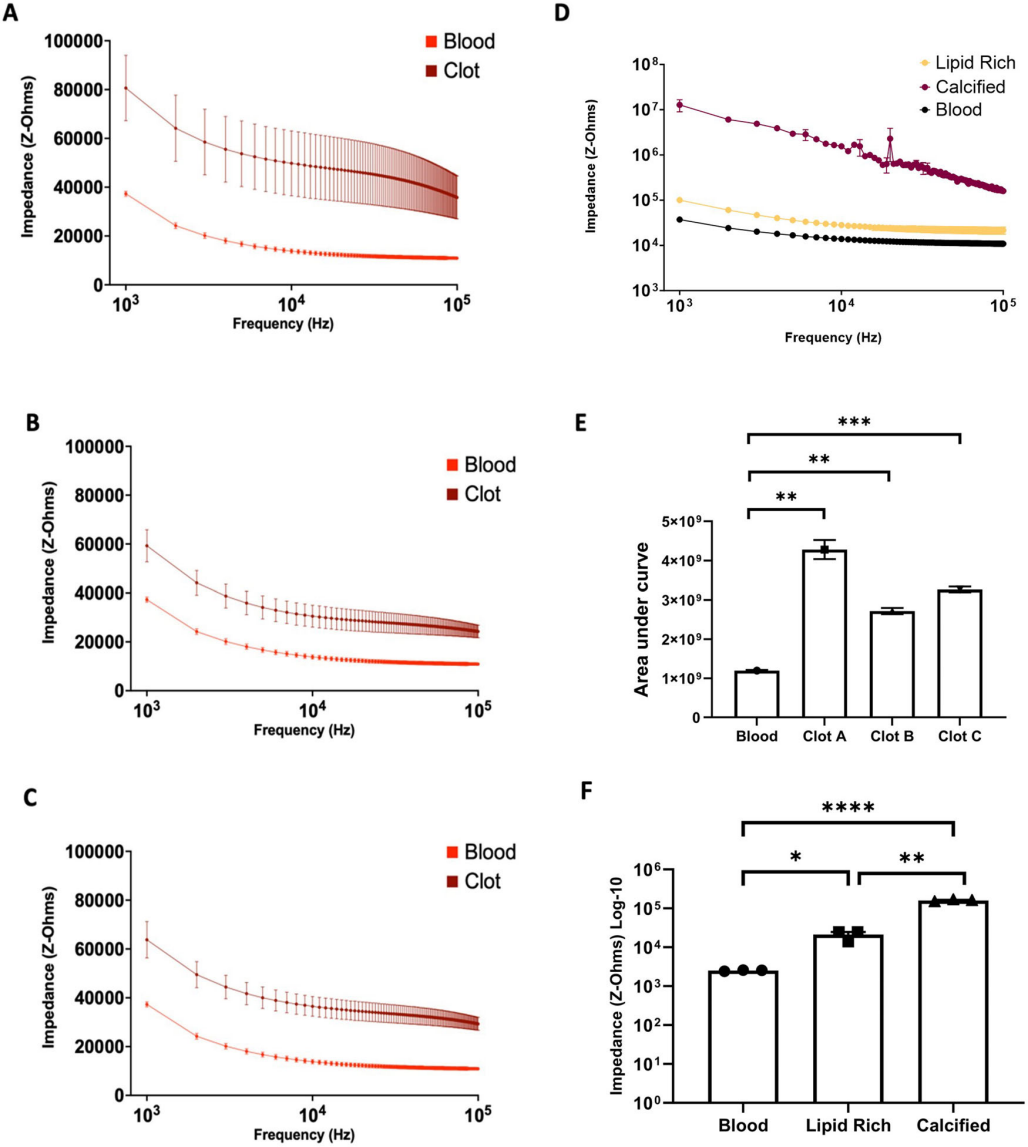
Comparison of blood clot and human plaque detection inside graft:-. Late-stage complications of graft failure. **A**–**C** Comparison of heparinised blood as control versus different blood clot of the same mass. **D** Human ex vivo plaque from carotid endarterectomy specimens representing cellular restenotic tissue detected; impedance of a lipid-rich region of explanted carotid artery versus calcified region. **E** Comparison of blood clots by area under the curve of the frequency spectrum mean ± SEM (*N* = 3 independent biological repeats), test degrees of freedom = 2. **E** Blood clot Area under the curve analysis. **F** Human plaque comparison of impedances at 100 kHz. Mean ± SEM (*N* = 3), *t*-test, degrees of freedom = 2. Blood vs. Clot A **p < 0.01, Blood vs. clot B **p < 0.01. Blood vs. clot C ***p < 0.001. Exact *p*-values are in the Supplementary Data.

The most common complication of AVG is thrombosis with VS implicated in the majority of events. This, in turn, has an immediate impact on emergency hospital treatment that may require alternative VA and thrombectomy and ultimately has a detrimental impact on AVG longevity. Therefore, in this study, we investigated how heparinised blood detection would differ from clotted blood on our sensor ex vivo. Three replicates of heparinised blood samples were compared to blood clots of equal mass and are presented in Fig. 5A–C. The frequency sweeps of the impedance shown for the heparinised blood and clotted blood are shown in Fig. 5A–C (1–100 kHz). Note, at all the frequency, points blood clots show a distinctive increase in impedance compared to blood. A distinctive and reproducible impedance signature was observed when comparing the area under the curve data for the three blood clots.

In the later stages of neointimal hyperplasia, the lesion of VSMC can become calcified. This initial hardening of the neo plaque leads to stabilizing the culprit lesion, possibly leading to a reduced risk of embolism. Conversely, if the plaque is not calcified but is instead composed of a lipid-rich constitution the risk of emboli increases. In Fig. 5E plaques were placed on the sensor and the same pair of electrodes was used to measure the impedance across the lipid-rich section and calcified region. Across the same frequency spectrum (1–100 kHz) a significant decrease in the impedance was observed between the calcified plaque and that of the lipid-rich one Fig. 5D. Discerning between the two types of complications is significant and potentially allows for a proportionate response to be taken based on the constitution of the blockage. For example, reducing the risk of dislodging emboli while providing both a quantitative and qualitative assessment.

**Fig. 6:**
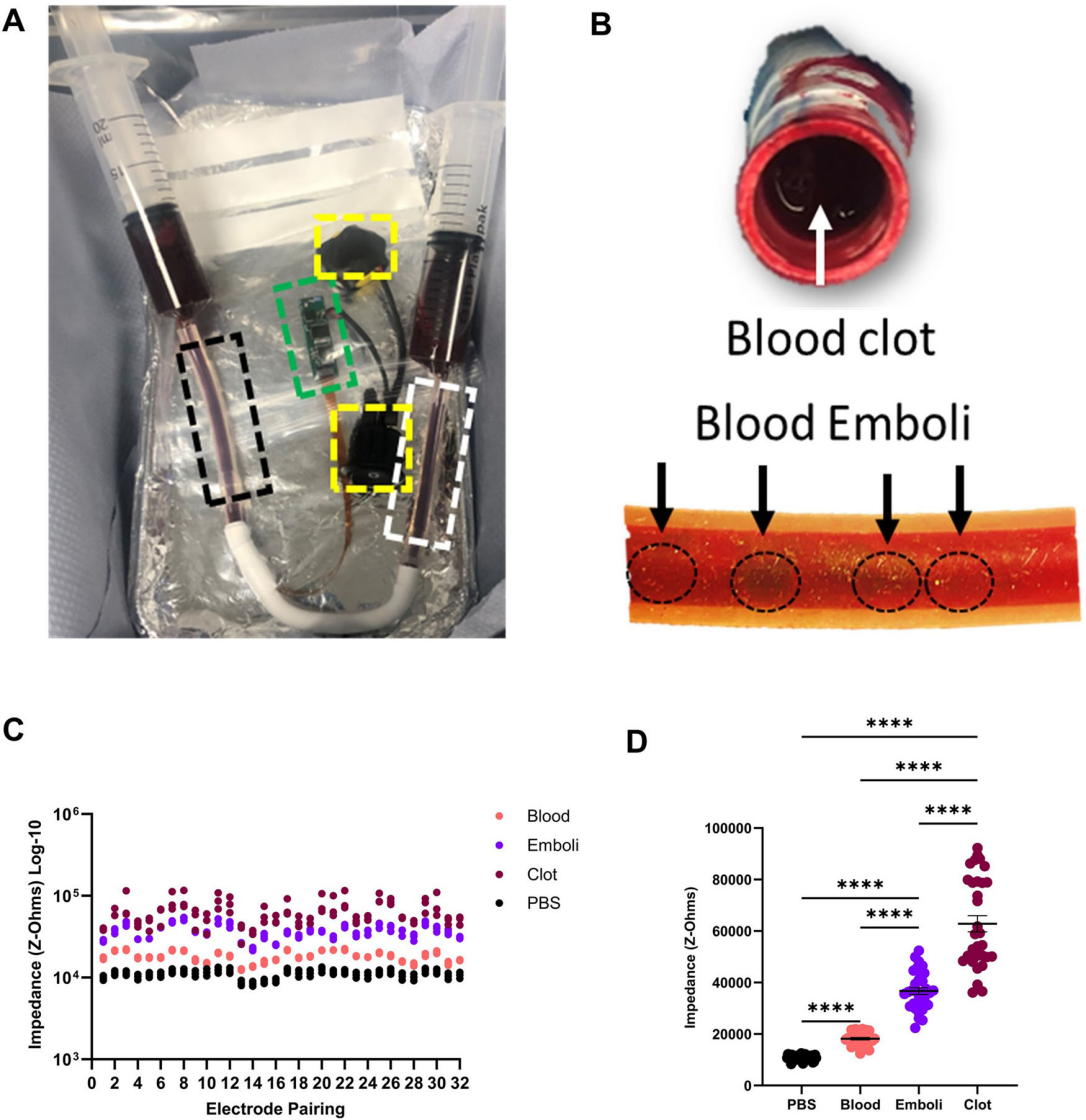
Prototype graft flow experiments. **A** Experimental setup of the flow experiment. Inflow-black, outflow-white, PCB-green, battery and switch in yellow box hatching. **B** Clot in situ inside of the graft (upper panel) and emboli flowing through inside of graft section (lower panel). **C** shows the impendence value of each pairing of electrodes. **D** shows all electrode parings grouped and tested using mean one-way ANOVA ± SEM (*N* = 3 independent biological repeats) *F* value = 129.8, degrees of freedom = 2 PBS vs. Blood, ****p < 0.0001, PBS vs. Emboli, ****p < 0.0001, Blood vs. Emboli ****p < 0.0001, PBS vs. Clot ****p < 0.0001, Blood vs. Clot ****p < 0.0001, Emboli vs. Clot ****p < 0.0001. Exact *p*-values are in the Supplementary Data, except for values where GraphPad Prism indicated that the *p* value was < 0.0001.

To test the effect of the blood clot and emboli across the implant was attached to 20 mL reservoir syringes and the air was removed from the line by flushing the device with heparinised porcine blood (see Fig. 6A). Once air bubbles were removed, blood was aspirated and impedance measurements were made as a control. The graft was then disconnected, and inlets were blocked. A porcine blood clot was dissected from a coagulated bloodstock and placed in the graft to occlude it, (see Fig. 6B). With the air removed once more, blood was refilled into the graft and measurements were made. To mimic emboli the blood reservoir in the syringe was mixed with micro-blood clots, this was achieved by aerating the clot in the presence of heparinised blood in a Petri dish. The measurements from the device are shown at 100 kHz for each sensor pairing, see Fig. 6C. The multiplexed device takes a total of 32 sensor readings created from two banks of 8 connections within the multiplexer. As such, 8 positive and 8 negative electrodes can be selected, and the impedance measured within them. A total of 64 possible combinations can be achieved by pairing the electrodes. Only 32 are used in our algorithm due to the symmetry of the vessel. In Fig. 6D the electrode pairs are shown for blood, an occluded vessel with clot and emboli. The blood has the lowest impedance followed by the emboli with the clot having the greatest impedance. Within this data, one observes the magnitude of the readings shift from electrode pairing to electrode pairing however the difference between blood–emboli–clot remains relatively similar for each pairing. This is due to the circumferential nature of the sensor and the pairing pattern where electrodes closer together have a lower impedance than those electrodes that are further apart. When the sensors are grouped by sample the difference between the experimental groups can be observed (see Fig. 6D). Here a significant difference is observed between all groups. The clot group shows a higher impedance group and a lower impedance group. When comparing this to the distance of electrodes those electrodes closer on the circumference are lower than those further apart. Whereby the lengthening of the path increases the baseline resistance as conductivity remains equal.

### Preclinical Investigations

The use of animals in research is highly regulated in the UK under the Animal Welfare Act 2006 and the NC3Rs. Swine is internationally recognized as the gold standard for surgical models of implantable medical devices. Our use of female swine was justified based on the reduced fat in the peri-surgical area that could attenuate the expected radiotelemetry signals. Swine are justified on anatomical, physiological and biochemical grounds. They provide the best prehuman method of evaluating technology with the minimum number of justified animals to obtain effective and statistically robust data before clinical trials. Before any preclinical work, the encapsulated device was implanted in an animal cadaver, under the authority of Home Office license PP1529920 and in compliance with the NC3Rs and ARRIVE guidelines.

**Fig. 7:**
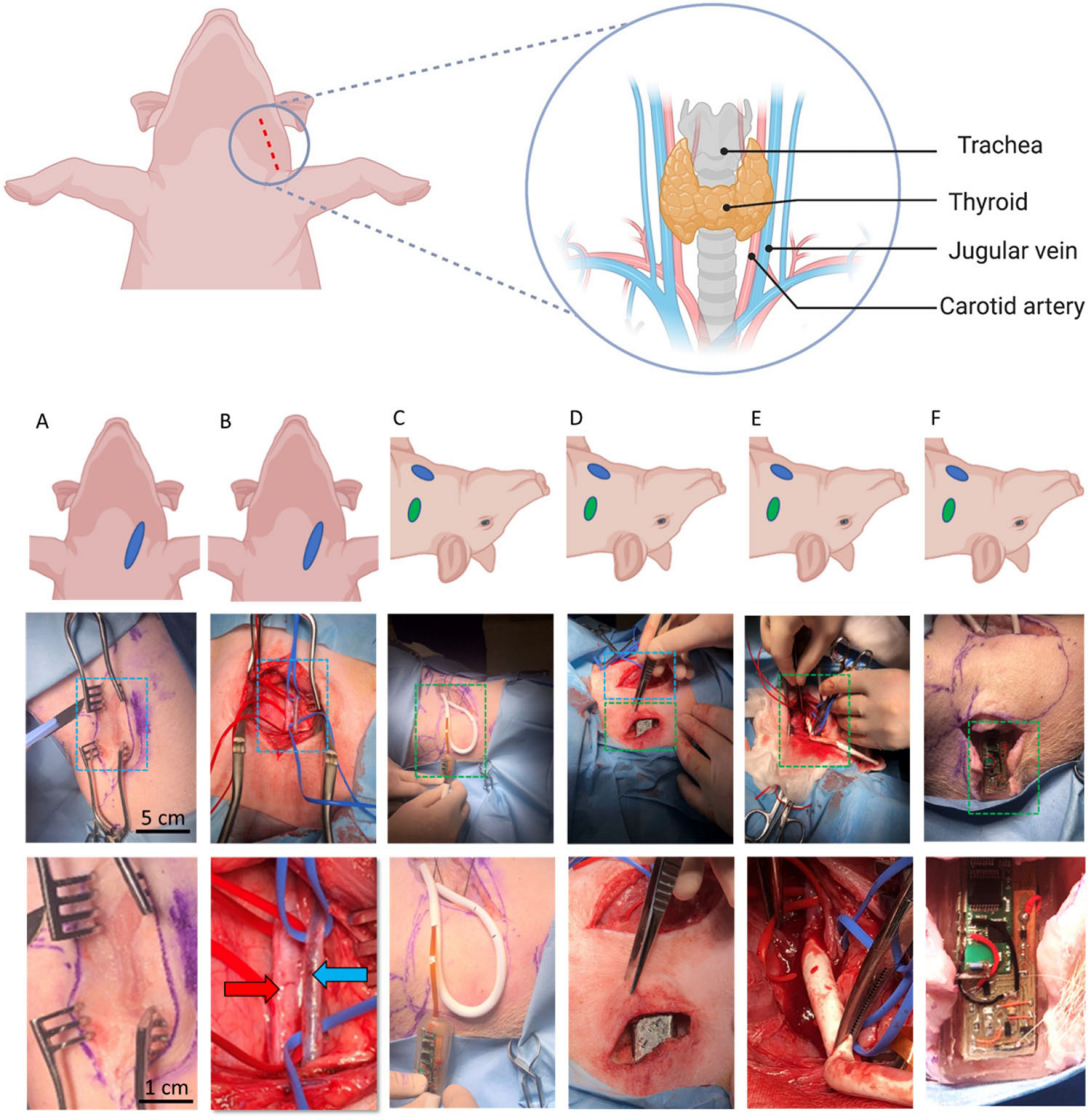
Surgical plan and technique for graft implantation. **A** Primary incision. **B** Exposure of the vessels—artery red arrow and vein blue arrow, sloops used to raise vessels up to surgical plain **C** sizing of looped AV graft for creation of side-pocket for radiotelemetry. **D** Biocompatible electronics package in situ. **E** AV graft in situ and sutured onto artery with graft clamped. **F** Wireless electronic impedance electronics activated and wireless relaying test impedimetric data before surgical closure.

Placed supine the midline was identified by locating the manubrium sterni. This was palpated about the shoulder joint to orientate and identify the sternohyoideus resting anterior to the oesophagus. The external jugular vein and carotid artery were then exposed through a retro-sternocleidomastoid exposure of the carotid sheath, with caution to avoid touching the hypoglossal and vagus nerves (Fig. 7A–F). A second incision was made for a small pocket for the wireless impedance electronics to be placed (see Fig. 7D). This was created through a second perpendicular incision ~7 cm from the first incision but on the side of the neck away from the mandible. The tether of the sensor was tunnelled through to the initial incision under the skin but over the device. The graft was then implanted as a shunt and anastomosed between the artery and vein with the sensor end joined to the venous side (see Fig. 7F). Cadaver (*n* = 1), non-recovery (*n* = 2).

The wireless impedance electronics unit is activated by applying an external magnetic field using a simple neodymium magnetic to the implant’s magnetically actuated power switch. Doing this then activates the device with a green light emitting diode (LED) to indicate that it has been powered on. It then immediately cycles through the sensor electrode pairings and transmits the raw impedance data to the transceiver connected to a laptop at a distance of 1 m away. The device remains active until the magnet is reapplied to deactivate the circuit. The transdermal magnetic switching was successfully activated above the sutured skin using this contactless method. This is positive for human implants in the forearm where the skin is much thinner and the adipose layer is often much reduced. In the clinical setting where dialysis canulation tubing can restrict the available area to activate the device, this is an important refinement compared to competitor wearable solutions. Note, that we observed the range of the encapsulated device on bench-top testing had a range of 10 m in the open air but as expected was attenuated to 1 m after surgery. This distance for relaying signals is more than sufficient due to the forearm being of a lower dimension than our animal model.

**Fig. 8:**
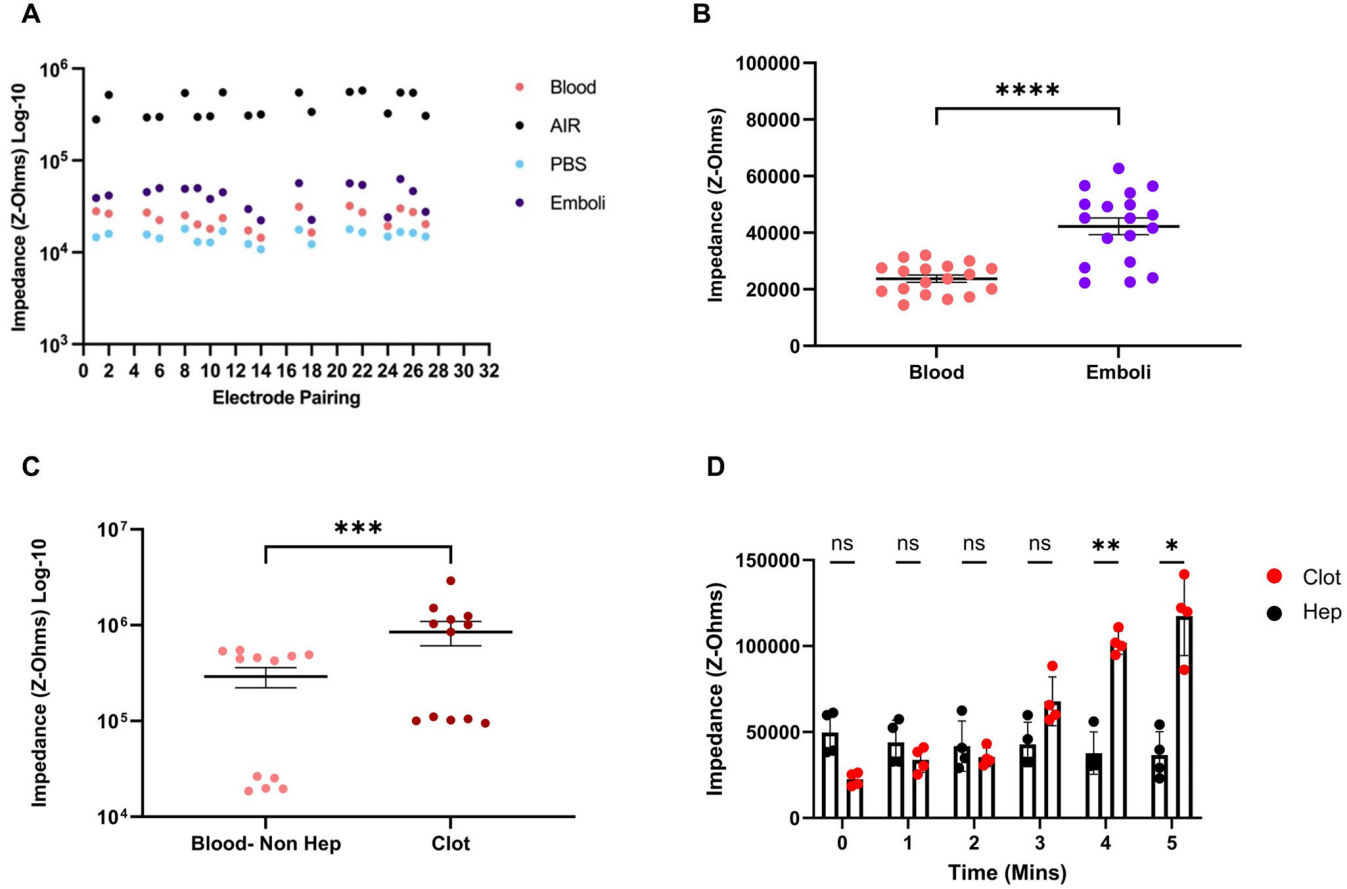
Impedance measurements from preclinical studies. **A** Impedance measurements across the sensors. **B** Shows the grouping of the repeats of blood and emboli (*N* = 18), Mean ± SEM, *t*-test, degrees of freedom (*n* = 17), ****p = <0.0001. **C** shows the difference from blood at T0 to clot at T5 (*N* = 12) Mean ± SEM, *t*-test, degrees of freedom = 11 ***p < 0.001. **D** Shows the graft clotting (clot) over time versus a heparinised graft (hep) (*N* = 4) mean ±SD. @ 4 min **p = <0.01, then at 5 min, *p* < 0.05. Exact *p*-values are in the Supplementary Data. 1x female cadaver for developing surgical approach, 2x female swine for non-recovery surgery sacrifice.

After completion of the surgery, a series of real-time measurements were made with different media within the graft when in situ. Figure 8A shows the impedance measurements from the electrodes, with the device in situ. No liquid shows air with the greatest impedance, PBS was the lowest and with blood and emboli higher respectively. Out of the possible 32 pairings, 18 electrodes that reported high-quality data were compared (see limitations). Figure 8B shows the comparison of the blood versus emboli, whereby emboli had a significantly higher impedance than blood. However, as the blood was artificially flowing a second model under physiological conditions was performed in non-recovery (*n* = 2), recovery (*n* = 2).

**Fig. 9:**
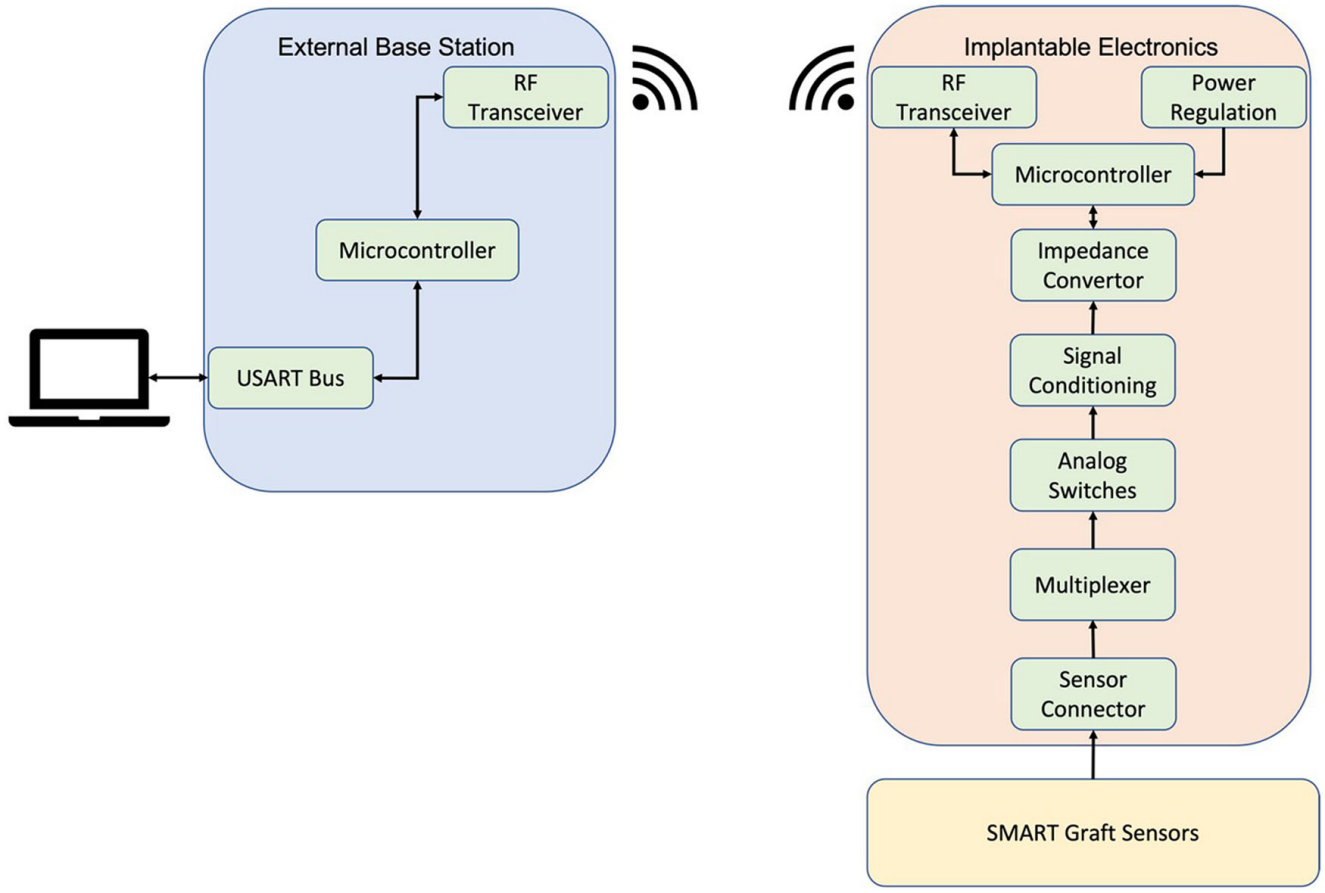
Diagram of base station and implantable electronics. Block diagram of the implantable electronics sensor input to data transmission from the implant to the base station. Here the data is transferred to the laptop where data is converted to a.csv file for analysis.

Serial measurements were made between a heparinised (negative control−100 units/kg of heparin) animal and a non-heparinised (positive control). Thrombus formation was induced by tying off the venous outflow track. Impedance changes were detected and tracked from inside the graft in real-time. A significant difference can be observed between normal blood flow and when the graft was occluded by a blood clot, (Fig. 8C). A time course of serial impedance readings was then made at 1 min intervals (Fig. 8D). The heparinised animal readings showed a modest decreasing trend in impedance across 0–5 min. In contrast, the non-heparinised animal by 3 min shows a clear inflexion point with the impedance increasing compared to the anticoagulated group. At 4 and 5 min the impedance of the antagonized animal was significantly different to the anticoagulated animal (*p* = 0.01, *p* = 0.05) (*n* = 2). Here the graft was implanted under physiological conditions and wireless impedance measurements were made via the implant and captured wirelessly outside the animal (Fig. 9).

## Discussion

Surveillance of AVG has been a source of controversy for many years, largely due to the failure of randomized control trials to support the rationale. This may have been due to poor trial design, inadequate or uncertain surveillance techniques and ineffectual interventions if VS is detected. Remote reporting of real-time data which may detect reduced flow would be an important development. Currently, routine surveillance is performed by 3 monthly ultrasound (US) scans to track flow through the AVG. A reduction of flow is an indicator of stenosis or a thrombotic event occurring. Whilst the US has been shown in robust observational series to improve outcomes, this necessitates repeated visits to a healthcare establishment. Given the frequent multi-morbidity and complexity of the healthcare needs of patients on haemodialyses, defaulting from surveillance is common, with an impact on the effectiveness of a surveillance programme.

The use of electrical impedance spectroscopy for determining the presence, adherence state and type of cell is well documented^25–27,34^. Previously our group has shown the use of impedimetric biosensors can be incorporated into existing medical devices such as coronary stents^37^. Moreover, our work has shown the detection between VSMCs, and endothelial cells. We propose that the device will create an overall impedance signature that is characteristic of vascular stenosis given the various cell types involved to be an early warning system for vascular stenosis. We accept that the threshold of clinically is yet to be determined. Here in Fig. 2, we demonstrated this within an innovative AV graft design^45^. This is in keeping with prior research from Holland et al. where the impedance spectra were used to differentiate cell types for in-stent restenosis^46^. Application of this technology for clinical use for a graft could be an appropriate solution for the early detection of venous stenosis and demonstrating clot detection in pre-clinical trials is a significant step for this field. Potentially this will allow for a close-looped remote telemetry system for the detection and treatment of premature AVgraft failure.

Post-operative remote wearable monitoring devices such as Alio, formally Graftworx, have shown potential benefits in the monitoring of patient vascular access. These have been shown to have reliability and feasibility with a recent trial reporting a sensitivity of 100% and specificity of 75% for <1 L/min flow rate identification in patient grafts^47^. In a separate study, researchers created a pressure sensor to monitor stenosis through the addition of a piezoelectric sensor wrapped in mylar on the outer of the graft^48^. Chong et al. showed an implantable version of a flow-based graft with a polydimethylsiloxane (PDMS) based external wraparound of the outer wall to act as a strain gauge sensor for pressure and flow detection^49^. The tethered electronics could wirelessly transmit data for a range of flows from 0 to 1.1 L/min with minimal deviation in pressure readout compared to commercially available pressure measuring equipment. Natta et al. 2019 presented a polyimide-PDMS-based piezoelectric micro-electromechanical systems (MEMs) sensor once more wrapped around the outer of the graft^50^. Validation through in vitro pulsatile flow showed the sensor detected 20%, 50%, and 80% occlusion in the grafts.

Here, we employed an entirely new methodology of sandwiching the sensor inside the wall of the expanded polytetrafluoroethylene (ePTFE) graft. By creating intraluminal sensing apertures that precisely aligned the sensors to apertures in the graft wall we created a 3D sensor array. This is unlike the prior sensing work, as here our sensors are in direct contact with the blood and other tissue but offer a cross-sectional profile no more intrusive than current stent struts. This allows for the direct detection of the earliest signs of occlusion. In the surgical model, we showed that no leakage was observed on suturing the graft in place for the testing nor did the sensor array that was bonded to the graft show signs of leakages when used under physiological pressures. Figure 2 supports that the earliest changes of graft occlusion through cellular adhesion and growth could be detected. While Fig. 8D confirms temporal clotting is detectable. The test clots are arterial in nature and may differ from those found in AV grafts. However, we have assumed they will be sufficiently similar in nature to have a higher impedance than blood as shown in in vivo. While chronic thrombus will also differ from newly formed clots the device is designed to detect the earliest signs of clot formation. However, longer-term animal models will be needed to confirm this. The benefits of our approach are the direct detection of the cellular buildup of a clot. This provides more clinically relevant information about the size and composition of the occlusion than previous methods. Indeed, the location of a single sensor in one plane could limit detection. As such multiple bands of circumferential sensors along the graft would allow more data to be gathered in conjunction with the temporal trends. However, circuit complexity, implant size and power consumption are always a concern when increasing the number of inputs and outputs of the device.

Tethered electronics is also a deployable solution, due to the similarities of a pacemaker’s extracardiac placement. For a prototype preclinical or even first in human trial efficacy such packaging has been established through the Stentrode technology from Synchron though this is in the low-pressure vascular system^51–53^. The ultimate goal is for an application-specific integrated circuit (ASIC) fully embedded into the graft wall to reduce the complexity of the implant procedure. A flow-based sensor device with inductive powering of the ASIC has already shown this is a viable solution^54^. However, this being a pressure device it only assesses later obstructive pathologies where our proposed device acts as an early warning system for cellular-level pathological events.

### Limitations and future considerations

We acknowledge that although this was performed while the animal was anesthetised and as such more data over time is needed to assess the device’s long-term potential. As mentioned, although the use of extra lumen wrapping is appropriate for grafts, the apertures have not been assessed. However, we did note the loss of some electrodes due to flexion of the polyimide electrodes losing continuity. We are actively pursuing improved sensor array technology that will overcome this issue. Computational fluid dynamics has also been performed, which shows minimal interaction, however, a more in-depth investigation should be performed and reported. While impedance alone shows significant evidence of the device’s functionality considerably more insight can be gained by measuring magnitude and phase separately. Currently, this is outside of the wireless impedance electronics source code but will be upgraded in future iterations to improve the sensitivity and fidelity of the system.

## Conclusion

Previously it has been shown that wearable biosensors can detect vascular complications but here we show the advantages of active implanted medical devices that can be used as diagnostic instruments. In this work, we have created and tested a multi-electrode array that is designed to detect the earliest restenosis and thrombosis in a vascular graft. Through pre-clinical trials we have shown that the sensor can detect blood clots and emboli in vivo and through our in vitro work, with cultured cells, we show a pathway for early stenosis detection for long-term implants. The advantages of our approach are that it gives a more direct measurement of the size and composition of the occlusion, information that previously was unobtainable. This will improve clinical decision-making, whilst offering the hopes and benefits of community-based healthcare monitoring.

### Supplementary information

Supplementary Data

Reporting Summary

Description of Additional Supplementary Files

## Data Availability

The numerical data underlying the graphs shown in Figs. 3, 5, 6 and 8 are in the Supplementary Data. All other data sets are available on request and kept on an accessible server. No previously published or shared data was used in this publication.
