# Supplementary material for: Realtime monitoring of thrombus formation in vivo using a self-reporting vascular access graft
Source: Commun Med (Lond). 2024 Feb 5;4:15. doi: 10.1038/s43856-024-00436-8 (PMC10844314; doi:10.1038/s43856-024-00436-8)
Supplement: Supplementary file 1 — Supplementary Data [file 43856_2024_436_MOESM1_ESM.pdf]

Supplemental Data

Figure 3

			D									
	Clot C		Freq		Lipid Rich			Calcified			Blood	
60200	53000	78100	1000	104000	112631	84600	15078300	5298560	17900000	35600	37100	39200
45500	42900	60100	2000	67200	63035	51500	5318050	6489350	6420000	22000	25700	25000
40200	39000	54000	3000	53100	46072	41700	4947180	4603030	5110000	17900	21600	21000
37500	36800	50800	4000	46000	37477	37100	3686230	3586070	4410000	15900	19300	19000
35800	35400	48800	5000	41600	32448	34400	2745310	3220920	2890000	14700	17800	17700
34800	34400	47500	6000	38600	28694	32600	4313370	2553630	1680000	13800	16700	16800
34000	33700	46500	7000	36600	26551	31600	2108150	2282310	2280000	13200	16000	16200
33400	33100	45700	8000	35100	24671	30600	1776440	1797140	1740000	12800	15400	15600
33000	32700	45100	9000	33900	23319	29900	1734750	1765320	1450000	12400	14900	15200
32600	32300	44600	10000	32900	22120	29300	1578140	1590250	1490000	12100	14500	14900
32300	31900	44200	11000	32200	21385	28200	1468080	987911	1190000	11900	14200	14600
32000	31700	43900	12000	31500	20656	28600	1350670	1875590	1820000	11700	13900	14400
31800	31400	43600	13000	31000	19878	28300	2751060	1248390	667000	11500	13700	14200
31600	31200	43300	14000	30600	19209	28100	879861	1061100	884000	11300	13500	14000
31400	31000	43100	15000	30100	18928	27500	859999	1046160	1080000	11200	13300	13800
31300	30900	42800	16000	29850	18299	26400	805155	948217	835000	11100	13100	13700
31100	30700	42600	17000	29470	18048	27500	735945	632236	913000	11000	13000	13600
31000	30500	42500	18000	29240	17576	27000	736617	554197	520000	10900	12900	13500
30900	30400	42300	19000	29050	18392	25300	902379	820766	166000	10800	12800	13400
30800	30300	42100	20000	28930	17459	26900	627344	797359	5450000	10800	12700	13300
30700	30200	42000	21000	28600	17011	26900	630350	638510	639000	10700	12600	13200
30600	30100	41900	22000	28450	16831	26400	590845	637343	725000	10600	12500	13100
30500	30000	41700	23000	28360	16798	26900	634827	918106	575000	10600	12400	13100
30500	29900	41600	24000	28180	16526	26200	573175	616610	610000	10500	12400	13000
30400	29800	41500	25000	28090	16321	26100	670597	719694	442000	10500	12300	13000
30300	29700	41300	26000	28010	16110	26000	586659	598499	674000	10500	12300	12900
30200	29600	41300	27000	27850	16281	25800	580011	543572	464000	10400	12200	12900
30200	29600	41100	28000	27810	16079	26500	549146	596773	682000	10400	12200	12800
30100	29500	41000	29000	27670	16134	25200	676868	823957	447000	10400	12100	12800
30100	29400	40900	30000	27590	16077	25800	517884	470819	556000	10300	12100	12700
30000	29400	40800	31000	27570	15816	26100	527107	558339	621000	10300	12000	12700
30000	29300	40700	32000	27390	15723	24800	418302	326539	829000	10300	12000	12700
29900	29200	40600	33000	27420	15537	25600	535048	476330	483000	10200	12000	12600
29900	29200	40500	34000	27290	15755	24500	389324	365554	800000	10200	11900	12600
29800	29100	40400	35000	27280	15450	25400	404995	455500	427000	10200	11900	12600
29800	29000	40300	36000	27220	15284	25600	422883	427832	349000	10200	11900	12600
29700	29000	40200	37000	27220	15231	25100	462394	496222	385000	10200	11900	12500
29700	28900	40100	38000	27170	15322	25400	361838	555216	401000	10100	11800	12500
29600	28900	40000	39000	27060	15052	25100	389473	480402	378000	10100	11800	12500
29600	28800	40000	40000	27060	15074	25300	399505	389541	358000	10100	11800	12500
29500	28800	39900	41000	27030	15162	25100	359387	403441	505000	10100	11800	12500
29500	28700	39800	42000	27000	15037	25400	370230	372217	342000	10100	11700	12400
29500	28700	39700	43000	26920	14896	25200	346308	332598	379000	10100	11700	12400
29400	28600	39600	44000	26920	15106	24700	366302	340530	469000	10000	11700	12400
29400	28600	39500	45000	26830	14843	25000	364309	366761	322000	10000	11700	12400
29300	28500	39400	46000	26820	14853	25300	354698	344271	357000	10000	11700	12400
29300	28500	39300	47000	26820	14770	25500	338491	336633	301000	10000	11600	12300
29300	28400	39200	48000	26800	14926	25300	310597	341133	310000	9990	11600	12300
29200	28400	39100	49000	26770	14704	24800	316935	334498	340000	9980	11600	12300
29200	28300	39100	50000	26760	14822	25000	331127	295210	326000	9970	11600	12300
29200	28300	39000	51000	26740	14901	25300	303948	307730	302000	9960	11600	12300
29100	28200	38900	52000	26670	14673	25200	275736	270296	228000	9950	11600	12300
29100	28200	38800	53000	26660	14660	24800	319401	278937	269000	9940	11600	12300
29000	28100	38700	54000	26630	14768	25200	262083	292877	306000	9930	11500	12200
29000	28100	38600	55000	26610	14773	24700	322545	299161	275000	9920	11500	12200
29000	28100	38500	56000	26570	14635	24800	295541	256647	307000	9910	11500	12200
28900	28000	38400	57000	26580	14483	24400	275164	277242	296000	9900	11500	12200
28900	28000	38300	58000	26510	14437	24500	289026	288834	251000	9890	11500	12200
28800	27900	38200	59000	26570	14525	25100	289199	267481	269000	9890	11500	12200
28800	27900	38200	60000	26520	14620	24300	249807	253141	246000	9880	11500	12200
28800	27800	38100	61000	26480	14665	24500	215606	268702	208000	9870	11500	12200
28700	27800	38000	62000	26550	14560	24900	249562	292444	256000	9860	11400	12200
28700	27700	37900	63000	26500	14483	24500	253857	246857	242000	9860	11400	12100
28700	27700	37800	64000	26470	14402	24200	235512	267214	236000	9850	11400	12100
28600	27600	37700	65000	26450	14499	25300	272776	237568	216000	9840	11400	12100
28600	27600	37600	66000	26410	14415	24500	240203	231688	245000	9830	11400	12100
28600	27600	37500	67000	26430	14270	25200	217958	273889	219000	9830	11400	12100
28500	27500	37400	68000	26380	14433	24600	227751	227262	228000	9820	11400	12100
28500	27500	37300	69000	26400	14427	24700	232804	230047	238000	9810	11400	12100
28500	27400	37300	70000	26430	14304	24600	247493	243147	226000	9810	11400	12100
28400	27400	37200	71000	26350	14541	24300	227291	229407	203000	9800	11400	12100
28400	27300	37100	72000	26360	14247	24100	220331	216202	207000	9800	11300	12100
28300	27300	37000	73000	26360	14290	24000	215118	242037	205000	9790	11300	12100
28300	27300	36900	74000	26350	14283	24900	220402	222652	216000	9790	11300	12000
28300	27200	36800	75000	26290	14339	24700	213773	222244	214000	9780	11300	12000
28200	27100	36700	76000	26300	14201	24700	219045	220299	200000	9770	11300	12000
28200	27100	36600	77000	26260	13970	23800	207593	196550	198000	9770	11300	12000
28100	27100	36500	78000	26350	14228	24400	199990	214917	205000	9760	11300	12000
28100	27000	36400	79000	26330	14021	24900	198204	201310	186000	9760	11300	12000
28100	27000	36400	80000	26270	14262	24300	188581	213173	192000	9750	11300	12000
28000	26900	36200	81000	26300	13956	24400	203561	187976	179000	9750	11300	12000
28000	26900	36200	82000	26330	14190	24100	190021	185083	178000	9740	11300	12000
28000	26800	36100	83000	26260	14032	24300	184971	198558	198000	9740	11300	12000
27900	26800	36000	84000	26260	14078	24200	184858	193944	196000	9730	11200	12000
27900	26700	35900	85000	26270	14183	24000	226741	220714	156000	9730	11200	12000
27900	26700	35800	86000	26230	14195	23500	191378	180031	160000	9720	11200	12000
27800	26700	35700	87000	26280	13933	24300	189969	186670	165000	9720	11200	11900
27800	26600	35600	88000	26240	13942	24200	189891	181901	183000	9710	11200	11900
27800	26600	35500	89000	26160	14134	23700	167920	191284	153000	9710	11200	11900
27700	26500	35400	90000	26210	14182	23600	194189	178806	159000	9710	11200	11900
27700	26500	35400	91000	26160	14064	24000	204293	149019	213000	9700	11200	11900
27600	26400	35300	92000	26220	14172	24100	166744	169121	163000	9700	11200	11900
27600	26400	35200	93000	26170	14024	23900	170807	172437	159000	9690	11200	11900
27600	26400	35100	94000	26160	14034	24500	165090	172935	169000	9690	11200	11900
27500	26300	35000	95000	26190	13934	24200	179472	172238	168000	9680	11200	11900
27500	26300	34900	96000	26200	14013	24600	162366	166934	165000	9680	11200	11900
27500	26200	34800	97000	26150	13957	24000	170366	167738	182000	9670	11200	11900
27400	26200	34700	98000	26150	13974	23800	168509	163661	157000	9670	11100	11900
27400	26100	34600	99000	26160	13667	24900	164350	164845	154000	9660	11100	11900
27300	26100	34500	100000	26160	14027	24800	150083	168572	161000	9660	11100	11900

Supplemental Data

Figure 5

E										F		
Freq	Control			MASMC			MEC			Control	MASMC	MEC
1000	17026.67	18546.67	20613.33	22700	19713.33	26840	16945.33	18880	18692	4170	12000	5880
2000	12618.67	12512	13886	18240.67	15433.33	21753.33	13044	14561.33	14042.67	3930	7180	4070
3000	10796	10227.33	11264	16345.33	13663.33	19600	11396	12803.33	12130	4010	11700	6730
4000	9712	8972.667	9831.333	15200	12651.33	18340	10420.67	11766	11008	3470	10400	5300
5000	8958	8161.333	8913.333	14428.67	11947.33	17420	9748.667	11060	10246.67	4190	6440	4630
6000	8384.667	7577.333	8261.333	13804	11424	16746.67	9264	10544.67	9691.333	3810	10200	4920
7000	7938.667	7142.667	7756	13374	11012	16216	8854.667	10130.67	9252.667	3440	8200	5390
8000	7570	6801.333	7356	12978.67	10672.67	15754.67	8555.333	9783.333	8887.333	3910	5360	4690
9000	7262.667	6516.667	7038.667	12686	10383.33	15403.33	8278.667	9523.333	8606.667	4010	8950	5370
10000	6998	6280.667	6772	12413.33	10156.67	15073.33	8054.667	9292.667	8372.667	3310	7010	5060
11000	6770	6073.333	6542.667	12200	9938.667	14835.33	7875.333	9074.667	8124.667	3800	4200	4550
12000	6566.667	5917.333	6346.667	12058	9830.667	14402.67	7692	8895.333	7964.667	4250	7750	5430
13000	6396.667	5749.333	6167.333	11798.67	9615.333	14358.67	7518.667	8726	7806.667	3200	6460	4550
14000	6238	5608	6008	11618.67	9438.667	14090.67	7403.333	8603.333	7667.333	4080	4760	5030
15000	6088.667	5502	5852	11356.67	9314.667	14004	7236	8460.667	7516	4100	6930	5120
16000	5968	5378.667	5722.667	11122.67	9200.667	13491.33	7214	8356	7324	3420	7090	5270
17000	5844.667	5276	5641.333	11159.33	9057.333	13591.33	7084	8219.333	7294	4090	6040	5510
18000	5748	5190	5534	11071.33	8972.667	13417.33	6969.333	8137.333	7200.667	3710	5810	4860
19000	5598	5114.667	5417.333	10944	8938	13223.33	6915.333	8026	7032.667	3590	7140	5940
20000	5554.667	5030.667	5346	10920	8796	13171.33	6790.667	7930	7032	4100	7300	6150
21000	5464.667	4960	5262	10760.67	8707.333	12994.67	6737.333	7846	6959.333	3580	5220	5070
22000	5392.667	4893.333	5194	10644.67	8618	12888	6667.333	7782	6864	3350	6970	6810
23000	5313.333	4837.333	5106.667	10606.67	8532	12832	6585.333	7720.667	6774	3750	6620	6810
24000	5238.667	4774.667	5041.333	10461.33	8445.333	12658	6537.333	7648	6734	3300	4720	5080
25000	5179.333	4718.667	4979.333	10416.67	8422.667	12512	6476	7596.667	6695.333	3330	7720	7050
26000	5124.667	4670.667	4919.333	10300.67	8319.333	12472.67	6442.667	7541.333	6619.333	3400	6070	7160
27000	5066	4610	4898	10188.67	8286	12418	6365.333	7462	6561.333	3050	4600	4390
28000	5014.667	4589.333	4819.333	10193.33	8209.333	12290.67	6329.333	7419.333	6512	3770	8120	6380
29000	4970.667	4542	4754	10072	8143.333	12285.33	6288	7376	6483.333	3590	6850	7840
30000	4924.667	4498.667	4721.333	9988.667	8129.333	12125.33	6234.667	7325.333	6428.667	3350	6290	4060
31000	4882	4449.333	4684	9967.333	8018.667	12008.67	6192	7280	6384.667	3830	9920	5070
32000	4840	4418	4635.333	9924	7962.667	11984	6170	7203.333	6318.667	3750	6920	6850
33000	4796	4380.667	4596	9792	7944.667	11914.67	6138.667	7170	6302	3500	6790	4130
34000	4753.333	4347.333	4561.333	9760.667	7926.667	11724.67	6075.333	7192	6279.333	3470	9030	4140
35000	4711.333	4323.333	4536.667	9754.667	7879.333	11668	6047.333	7091.333	6240	3900	7650	6710
36000	4688	4286	4498	9696.667	7802.667	11678	6028.667	7052	6210.667	3230	6860	3620
37000	4642	4270.667	4458.667	9664.667	7785.333	11512.67	5992	7029.333	6180	3430	7040	3880
38000	4621.333	4232.667	4440.667	9562.667	7714	11482	5957.333	6982.667	6163.333	3940	9700	6640
39000	4584	4202	4392.667	9494.667	7690.667	11414	5936.667	6969.333	6094	3140	7820	3550
40000	4564	4186.667	4367.333	9496.667	7643.333	11394.67	5902.667	6935.333	6081.333	3800	8790	3990
41000	4532.667	4160	4336	9424.667	7646.667	11238.67	5862.667	6901.333	6061.333	3820	10500	6700
42000	4510.667	4142	4318	9422	7580	11200	5859.333	6863.333	6021.333	3360	8600	4430
43000	4478	4118.667	4294	9334.667	7563.333	11206	5838.667	6827.333	6009.333	3850	12200	3890
44000	4466.667	4092.667	4265.333	9308	7519.333	11121.33	5797.333	6837.333	5976	4240	11200	8850
45000	4444	4077.333	4244.667	9284.667	7492	11036	5782.667	6778	5946	3490	10100	6080
46000	4410	4058.667	4217.333	9189.333	7459.333	10979.33	5770	6737.333	5950.667			
47000	4390.667	4036	4205.333	9195.333	7407.333	10936	5738	6719.333	5904.667			
48000	4374.667	4024	4182	9152	7386	10878.67	5726.667	6695.333	5896.667			
49000	4344	3994	4146.667	9107.333	7385.333	10840.67	5701.333	6682.667	5874			
50000	4331.333	3981.333	4138.667	9060.667	7354.667	10772.67	5683.333	6661.333	5846			
51000	4317.333	3973.333	4133.333	9048	7322.667	10734.67	5676.667	6639.333	5832			
52000	4301.333	3950.667	4100	9033.333	7252	10664.67	5640.667	6616.667	5808.667			
53000	4271.333	3942	4074.667	8961.333	7228.667	10634.67	5639.333	6590.667	5787.333			
54000	4262	3920.667	4072	8937.333	7247.333	10559.33	5612.667	6573.333	5771.333			
55000	4250	3913.333	4050	8918	7208	10548	5599.333	6548	5742.667			
56000	4228.667	3897.333	4040.667	8856	7198	10527.33	5573.333	6531.333	5729.333			
57000	4218	3882	4020.667	8802	7169.333	10484	5550.667	6506	5711.333			
58000	4199.333	3873.333	4016	8833.333	7164.667	10408.67	5506.667	6478.667	5708.667			
59000	4194	3848.667	3995.333	8714	7100	10330.67	5526	6474	5687.333			
60000	4166.667	3848.667	3960.667	8690	7094	10290	5496.667	6460	5659.333			
61000	4160	3832.667	3972	8664.667	7092.667	10268	5492.667	6445.333	5665.333			
62000	4138	3822	3936.667	8745.333	7065.333	10214.67	5490.667	6419.333	5660			
63000	4139.333	3816.667	3942	8645.333	7023.333	10208.67	5470	6401.333	5614.667			
64000	4118	3800	3922.667	8616.667	7003.333	10167.33	5460	6370.667	5610			
65000	4114	3791.333	3914	8612.667	6984.667	10123.33	5445.333	6359.333	5594.667			
66000	4101.333	3774	3904	8579.333	6980	10098	5445.333	6339.333	5588.667			
67000	4084	3766	3880.667	8508.667	6961.333	10053.33	5432.667	6343.333	5563.333			
68000	4082.667	3759.333	3881.333	8501.333	6954	10000.67	5413.333	6312.667	5564			
69000	4070.667	3755.333	3869.333	8485.333	6935.333	9967.333	5406	6290	5540			
70000	4065.333	3732.667	3866	8452	6888.667	9965.333	5393.333	6268	5549.333			
71000	4049.333	3729.333	3849.333	8360	6883.333	9893.333	5365.333	6262.667	5510			
72000	4038	3723.333	3836	8400	6844.667	9852	5365.333	6253.333	5515.333			
73000	4030.667	3718.667	3830	8418	6837.333	9891.333	5337.333	6242	5500			
74000	4015.333	3710	3822	8360	6828.667	9808	5344.667	6231.333	5486			
75000	4008	3706.667	3818.667	8344.667	6788.667	9740.667	5344.667	6212	5476.667			
76000	4008.667	3694.667	3794.667	8278	6808.667	9709.333	5315.333	6200	5472			
77000	3988	3686.667	3806	8353.333	6736.667	9720.667	5304.667	6144	5447.333			
78000	3970	3648.667	3778.667	8308	6767.333	9630.667	5295.333	6167.333	5432.667			
79000	3988	3677.333	3782.667	8240	6716	9616.667	5316	6191.333	5454			
80000	3980.667	3676.667	3767.333	8230	6697.333	9621.333	5266.667	6150.667	5406			
81000	3966	3653.333	3758	8210.667	6722	9584	5284	6120.667	5401.333			
82000	3954	3656.667	3748	8164.667	6668	9532.667	5267.333	6130.667	5417.333			
83000	3948	3650	3741.333	8148	6684.667	9492.667	5258.667	6121.333	5396.667			
84000	3940	3638	3744.667	8156	6682.667	9464	5253.333	6100	5386			
85000	3933.333	3633.333	3726	8110	6640.667	9448	5251.333	6120	5356			
86000	3937.333	3641.333	3706.667	8002	6582	9380.667	5234	6098	5338			
87000	3927.333	3612.667	3738.667	8130	6606.667	9370	5238.667	6056.667	5345.333			
88000	3907.333	3615.333	3716.667	8048.667	6618	9363.333	5218.667	6064	5346			
89000	3909.333	3606	3708.667	8032.667	6590.667	9323.333	5200	6030	5346.667			
90000	3893.333	3597.333	3702	8022	6613.333	9282	5198	6027.333	5333.333			
91000	3883.333	3598	3688.667	8006	6543.333	9260	5190.667	6020.667	5292			
92000	3879.333	3589.333	3682.667	7980.667	6548.667	9248.667	5182.667	6012	5317.333			
93000	3884	3582.667	3686.667	7940	6554	9225.333	5184	5994	5320.667			
94000	3882	3583.333	3670	7944	6531.333	9164.667	5168	5992	5294.667			
95000	3866	3570.667	3669.333	7957.333	6510.667	9168	5168	5989.333	5295.333			
96000	3866.667	3577.333	3666.667	7881.333	6505.333	9147.333	5162	5969.333	5288			
97000	3857.333	3564	3657.333	7908.667	6498	9116	5154.667	5959.333	5275.333			
98000	3852.667	3558.667	3653.333	7868	6477.333	9035.333	5144.667	5966.667	5280.667			
99000	3868.667	3554	3621.333	7995.333	6511.333	8975.333	5112	5932	5244.667			
100000	3845.333	3558.667	3650	7836	6437.333	8874.667	5114.667	5892	5235.333			

Supplemental Data

Figure 6

			D									
Clot C			Freq	Lipid Rich			Calcified			Blood		
60200	53000	78100	1000	104000	112631	84600	15078300	5298560	17900000	35600	37100	39200
45500	42900	60100	2000	67200	63035	51500	5318050	6489350	6420000	22000	25700	25000
40200	39000	54000	3000	53100	46072	41700	4947180	4603030	5110000	17900	21600	21000
37500	36800	50800	4000	46000	37477	37100	3686230	3586070	4410000	15900	19300	19000
35800	35400	48800	5000	41600	32448	34400	2745310	3220920	2890000	14700	17800	17700
34800	34400	47500	6000	38600	28694	32600	4313370	2553630	1680000	13800	16700	16800
34000	33700	46500	7000	36600	26551	31600	2108150	2282310	2280000	13200	16000	16200
33400	33100	45700	8000	35100	24671	30600	1776440	1797140	1740000	12800	15400	15600
33000	32700	45100	9000	33900	23319	29900	1734750	1765320	1450000	12400	14900	15200
32600	32300	44600	10000	32900	22120	29300	1578140	1590250	1490000	12100	14500	14900
32300	31900	44200	11000	32200	21385	28200	1468080	987911	1190000	11900	14200	14600
32000	31700	43900	12000	31500	20656	28600	1350670	1875590	1820000	11700	13900	14400
31800	31400	43600	13000	31000	19878	28300	2751060	1248390	667000	11500	13700	14200
31600	31200	43300	14000	30600	19209	28100	879861	1061100	884000	11300	13500	14000
31400	31000	43100	15000	30100	18928	27500	859999	1046160	1080000	11200	13300	13800
31300	30900	42800	16000	29850	18299	26400	805155	948217	835000	11100	13100	13700
31100	30700	42600	17000	29470	18048	27500	735945	632236	913000	11000	13000	13600
31000	30500	42500	18000	29240	17576	27000	736617	554197	520000	10900	12900	13500
30900	30400	42300	19000	29050	18392	25300	902379	820766	166000	10800	12800	13400
30800	30300	42100	20000	28930	17459	26900	627344	797359	5450000	10800	12700	13300
30700	30200	42000	21000	28600	17011	26900	630350	638510	639000	10700	12600	13200
30600	30100	41900	22000	28450	16831	26400	590845	637343	725000	10600	12500	13100
30500	30000	41700	23000	28360	16798	26900	634827	918106	575000	10600	12400	13100
30500	29900	41600	24000	28180	16526	26200	573175	616610	610000	10500	12400	13000
30400	29800	41500	25000	28090	16321	26100	670597	719694	442000	10500	12300	13000
30300	29700	41300	26000	28010	16110	26000	586659	598499	674000	10500	12300	12900
30200	29600	41300	27000	27850	16281	25800	580011	543572	464000	10400	12200	12900
30200	29600	41100	28000	27810	16079	26500	549146	596773	682000	10400	12200	12800
30100	29500	41000	29000	27670	16134	25200	676868	823957	447000	10400	12100	12800
30100	29400	40900	30000	27590	16077	25800	517884	470819	556000	10300	12100	12700
30000	29400	40800	31000	27570	15816	26100	527107	558339	621000	10300	12000	12700
30000	29300	40700	32000	27390	15723	24800	418302	326539	829000	10300	12000	12700
29900	29200	40600	33000	27420	15537	25600	535048	476330	483000	10200	12000	12600
29900	29200	40500	34000	27290	15755	24500	389324	365554	800000	10200	11900	12600
29800	29100	40400	35000	27280	15450	25400	404995	455500	427000	10200	11900	12600
29800	29000	40300	36000	27220	15284	25600	422883	427832	349000	10200	11900	12600
29700	29000	40200	37000	27220	15231	25100	462394	496222	385000	10200	11900	12500
29700	28900	40100	38000	27170	15322	25400	361838	555216	401000	10100	11800	12500
29600	28900	40000	39000	27060	15052	25100	389473	480402	378000	10100	11800	12500
29600	28800	40000	40000	27060	15074	25300	399505	389541	358000	10100	11800	12500
29500	28800	39900	41000	27030	15162	25100	359387	403441	505000	10100	11800	12500
29500	28700	39800	42000	27000	15037	25400	370230	372217	342000	10100	11700	12400
29500	28700	39700	43000	26920	14896	25200	346308	332598	379000	10100	11700	12400
29400	28600	39600	44000	26920	15106	24700	366302	340530	469000	10000	11700	12400
29400	28600	39500	45000	26830	14843	25000	364309	366761	322000	10000	11700	12400
29300	28500	39400	46000	26820	14853	25300	354698	344271	357000	10000	11700	12400
29300	28500	39300	47000	26820	14770	25500	338491	336633	301000	10000	11600	12300
29300	28400	39200	48000	26800	14926	25300	310597	341133	310000	9990	11600	12300
29200	28400	39100	49000	26770	14704	24800	316935	334498	340000	9980	11600	12300
29200	28300	39100	50000	26760	14822	25000	331127	295210	326000	9970	11600	12300
29200	28300	39000	51000	26740	14901	25300	303948	307730	302000	9960	11600	12300
29100	28200	38900	52000	26670	14673	25200	275736	270296	228000	9950	11600	12300
29100	28200	38800	53000	26660	14660	24800	319401	278937	269000	9940	11600	12300
29000	28100	38700	54000	26630	14768	25200	262083	292877	306000	9930	11500	12200
29000	28100	38600	55000	26610	14773	24700	322545	299161	275000	9920	11500	12200
29000	28100	38500	56000	26570	14635	24800	295541	256647	307000	9910	11500	12200
28900	28000	38400	57000	26580	14483	24400	275164	277242	296000	9900	11500	12200
28900	28000	38300	58000	26510	14437	24500	289026	288834	251000	9890	11500	12200
28800	27900	38200	59000	26570	14525	25100	289199	267481	269000	9890	11500	12200
28800	27900	38200	60000	26520	14620	24300	249807	253141	246000	9880	11500	12200
28800	27800	38100	61000	26480	14665	24500	215606	268702	208000	9870	11500	12200
28700	27800	38000	62000	26550	14560	24900	249562	292444	256000	9860	11400	12200
28700	27700	37900	63000	26500	14483	24500	253857	246857	242000	9860	11400	12100
28700	27700	37800	64000	26470	14402	24200	235512	267214	236000	9850	11400	12100
28600	27600	37700	65000	26450	14499	25300	272776	237568	216000	9840	11400	12100
28600	27600	37600	66000	26410	14415	24500	240203	231688	245000	9830	11400	12100
28600	27600	37500	67000	26430	14270	25200	217958	273889	219000	9830	11400	12100
28500	27500	37400	68000	26380	14433	24600	227751	227262	228000	9820	11400	12100
28500	27500	37300	69000	26400	14427	24700	232804	230047	238000	9810	11400	12100
28500	27400	37300	70000	26430	14304	24600	247493	243147	226000	9810	11400	12100
28400	27400	37200	71000	26350	14541	24300	227291	229407	203000	9800	11400	12100
28400	27300	37100	72000	26360	14247	24100	220331	216202	207000	9800	11300	12100
28300	27300	37000	73000	26360	14290	24000	215118	242037	205000	9790	11300	12100
28300	27300	36900	74000	26350	14283	24900	220402	222652	216000	9790	11300	12000
28300	27200	36800	75000	26290	14339	24700	213773	222244	214000	9780	11300	12000
28200	27100	36700	76000	26300	14201	24700	219045	220299	200000	9770	11300	12000
28200	27100	36600	77000	26260	13970	23800	207593	196550	198000	9770	11300	12000
28100	27100	36500	78000	26350	14228	24400	199990	214917	205000	9760	11300	12000
28100	27000	36400	79000	26330	14021	24900	198204	201310	186000	9760	11300	12000
28100	27000	36400	80000	26270	14262	24300	188581	213173	192000	9750	11300	12000
28000	26900	36200	81000	26300	13956	24400	203561	187976	179000	9750	11300	12000
28000	26900	36200	82000	26330	14190	24100	190021	185083	178000	9740	11300	12000
28000	26800	36100	83000	26260	14032	24300	184971	198558	198000	9740	11300	12000
27900	26800	36000	84000	26260	14078	24200	184858	193944	196000	9730	11200	12000
27900	26700	35900	85000	26270	14183	24000	226741	220714	156000	9730	11200	12000
27900	26700	35800	86000	26230	14195	23500	191378	180031	160000	9720	11200	12000
27800	26700	35700	87000	26280	13933	24300	189969	186670	165000	9720	11200	11900
27800	26600	35600	88000	26240	13942	24200	189891	181901	183000	9710	11200	11900
27800	26600	35500	89000	26160	14134	23700	167920	191284	153000	9710	11200	11900
27700	26500	35400	90000	26210	14182	23600	194189	178806	159000	9710	11200	11900
27700	26500	35400	91000	26160	14064	24000	204293	149019	213000	9700	11200	11900
27600	26400	35300	92000	26220	14172	24100	166744	169121	163000	9700	11200	11900
27600	26400	35200	93000	26170	14024	23900	170807	172437	159000	9690	11200	11900
27600	26400	35100	94000	26160	14034	24500	165090	172935	169000	9690	11200	11900
27500	26300	35000	95000	26190	13934	24200	179472	172238	168000	9680	11200	11900
27500	26300	34900	96000	26200	14013	24600	162366	166934	165000	9680	11200	11900
27500	26200	34800	97000	26150	13957	24000	170366	167738	182000	9670	11200	11900
27400	26200	34700	98000	26150	13974	23800	168509	163661	157000	9670	11100	11900
27400	26100	34600	99000	26160	13667	24900	164350	164845	154000	9660	11100	11900
27300	26100	34500	100000	26160	14027	24800	150083	168572	161000	9660	11100	11900

Freq	E												F		
	Blood			Clot A			Clot B			Clot C			Blood	Lipid Rich	Calcified
1000	1.195E+09	8953478	3	4.279E+09	140323777	3	2.714E+09	45116054	3	3.262E+09	42784762	3	2425.85	26160	150083
2000													2552.49	14027.4	168572
3000													2591.89	24800	161000

Figure 8

A-B				C		D									
Blood	AIR	PBS	Emboli	Blood	Clot	T	Clot				Hep				
28163.365	279064.2	14552.594	38962.656	19566.63	104912.74	0	18537.873	25295.3	26359.819	19811.326	59806.676	39210.159	38282.171	61206.212	61206.212
26402.047	516989.16	15872.902	41627.315	18537.873	94781.832	1	25301.117	41058.647	38471.071	30303.391	52424.485	32775.727	32825.077	57482.87	57482.87
27122.717	293460.23	15626.295	45209.345	424258.6	1006610.5	2	30520.94	43182.071	34480.249	33421.847	62456.425	34762.61	29044.232	40828.144	40828.144
22463.18	298261.04	14197.498	49841.756	25295.3	110894.83	3	57211.222	65658.492	88487.269	60036.624	59772.872	32637.708	32739.461	45855.231	45855.231
25284.158	542939.82	18075.283	49213.506	536369.42	1141774.7	4	94781.832	110894.83	101993.18	100100.46	56099.348	30640.302	30263.46	33954.651	33954.651
20086.62	296931.42	12965.286	50070.132	26359.819	101993.18	5	119889.75	141668.64	86188.874	122151.43	54294.406	29235.214	23075.034	39713.172	39713.172
18031.212	303224.54	12859.038	38062.348	548192.76	1508350.3										
23696.166	549642.52	17055.596	45117.721	19811.326	100100.46										
17328.835	307402.3	12324.201	29632.683	491654.56	1241880.5										
14432.238	317056.52	10818.83	22324.249	445213.36	1031633.6										
31342.61	549485.52	17613.943	56611.692	474059.85	850159.28										
16459.495	337168.56	12239.38	22561.571	458659.66	2910487										
32072.459	556512.79	17824.561	56433.456												
27298.759	577768.05	16533.132	54015.902												
19322.056	323656.52	14835.348	24015.459												
30062.423	549485.52	16665.459	62748.916												
27510.069	546239.78	16308.083	46277.872												
20244.032	306381.58	14869.132	27666.043												

p-values

6D	
PBS vs. Blood	<0.0001
PBS vs. Clot	<0.0001
PBS vs. Emboli	<0.0001
Blood vs. Clot	<0.0001
Blood vs. Emboli	<0.0001
Clot vs. Emboli	<0.0001
8B	
Blood vs. Emboli	<0.0001
8C	
Blood-Non Hep vs. Clot	0.0005
8D	
T0	0.2367
T1	0.2356
T2	0.4616
T3	0.2367
T4	0.004
T5	0.02
