# Supplementary material for: Realtime monitoring of thrombus formation in vivo using a self-reporting vascular access graft
Source: Commun Med (Lond). 2024 Feb 5;4:15. doi: 10.1038/s43856-024-00436-8 (PMC10844314; doi:10.1038/s43856-024-00436-8)
Supplement: Supplementary file 3 — Description of Additional Supplementary Files [file 43856_2024_436_MOESM3_ESM.docx]

**Description of Additional Supplementary Files**

**File Name:** Supplementary Data

**Description:** Measurements taken from distinct biological samples as indicated. Inclusion of raw csv data used to generate all figure graphs including exact p-values obtained from statistical test.
